# Implementing the Blowers–Masel Approximation to Scale Activation Energy Based on Reaction Enthalpy in Mean-Field Microkinetic Modeling for Catalytic Methane Partial Oxidation

**DOI:** 10.1021/acscatal.3c05436

**Published:** 2024-05-09

**Authors:** Chao Xu, Emily J. Mazeau, Richard H. West

**Affiliations:** Department of Chemical Engineering, Northeastern University, Boston, Massachusetts 02115, United States

**Keywords:** mean-field microkinetic modeling, Blowers–Masel approximation, sensitivity analyses, linear scaling, catalytic methane partial oxidation

## Abstract

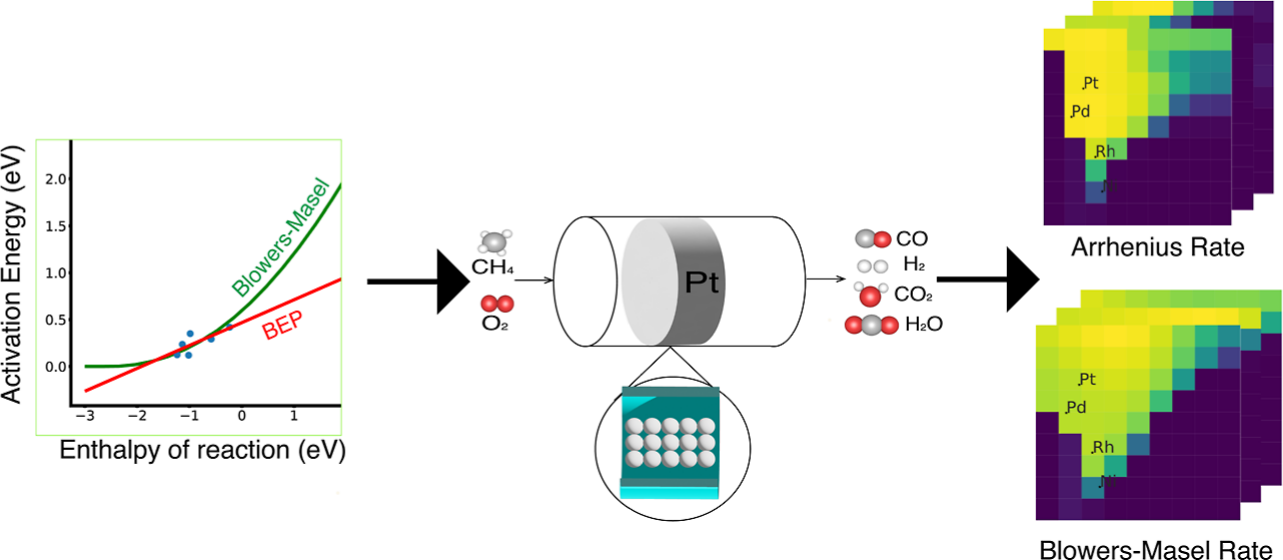

Mean-field microkinetic modeling is a powerful tool for catalyst design and the simulation of catalytic processes. The reaction enthalpies in a microkinetic model often need to be adjusted when changing species’ binding energies to model different catalysts, when performing thermodynamic sensitivity analyses, and when fitting experimental data. When altering reaction enthalpies, the activation energies should also be reasonably altered to ensure realistic reaction rates. The Blowers–Masel approximation (BMA) relates the reaction barrier to the reaction enthalpy. Unlike the Brønsted–Evans–Polani relationship, the BMA requires less data because only one parameter, the intrinsic activation energy, needs to be determined. We validate this application of BMA relations to model surface reactions by comparing against density functional theory data taken from the literature. By incorporating the BMA rate description into the open-source Cantera software, we enable a new workflow, demonstrated herein, allowing rapid screening of catalysts using linear scaling relationships and BMA kinetics within the process simulation software. For demonstration purposes, a catalyst screening for catalytic methane partial oxidation on 81 hypothetical metals is conducted. We compared the results with and without BMA-corrected rates. The heat maps of various descriptors (e.g., CH_4_ conversion, syngas yield) show that using BMA rates instead of Arrhenius rates (with constant activation energies) changes which metals are most active. Heat maps of sensitivity analyses can help identify which reactions or species are the most influential in shaping the descriptor map patterns. Our findings indicate that while using BMA-adjusted rates did not markedly affect the most sensitive reactions, it did change the most influential species.

## 1 Introduction

Heterogeneous catalysis plays a crucial role in the production of 80% of chemical products worldwide, and the catalyst market is expected to grow by 4.4% annually from 2020 to 2027.^1^ Designing efficient and cost-effective catalysts necessitates an understanding of the underlying mechanism. This knowledge enables the optimization of catalyst morphology and reaction conditions, such as temperature and pressure, to improve the catalyst performance. By manipulation of the active sites and reaction conditions based on the reaction mechanism, catalysts can be designed to achieve higher levels of activity, selectivity, and stability. Mean-field microkinetic modeling (MKM) has proven to be a powerful tool to identify and interpret intermediates and reactions in processes such as gas-phase combustion^2,3^ and catalysis^4−6^ and has been widely used for catalyst optimization. As demonstrated by its widespread and growing use,^7^ MKM has the potential to help discover and design new catalysts to support critical industrial processes.^8,9^

Furthermore, the linear scaling relationships (LSRs) developed by Abild-Pedersen et al.^10^ enhance the utility of MKM without excessive computational cost by creating a fast and simple way to predict the binding energy of surface species on different metal surfaces by using the adsorption energy of a species on one metal and scaling it to any other metal. While density functional theory (DFT) calculations are commonly used to compute the binding energies of surface species, performing DFT calculations for species on a large number of metals is computationally expensive. Consequently, LSRs are a useful approximation for rapidly estimating species’ thermodynamic properties and screening potential catalysts.

It is important to note that adjusting species’ enthalpies using linear scaling relationships can alter reaction enthalpies, necessitating the recalculation of transition states to ensure realistic reaction rates. As a substitute for DFT, and to reduce computational costs, the Brønsted–Evans–Polanyi (BEP) relationship,^11,12^ a linear relationship between the reaction enthalpy and activation energy, is commonly employed in published works.^13−15^ As discussed by Abild-Pedersen et al.,^10^ a preliminary catalyst screening can be done by acquiring an estimate of the full energy diagram of surface reactions on a range of catalysts with linear scaling and BEP relations. This could be followed by DFT calculations or experiments on any promising catalyst found in the screening.

However, BEP parameters are not easy to derive due to the scarcity of thermodynamic and kinetic data for surface reactions. Furthermore, Blowers and Masel^16^ pointed out that for certain reaction families, such as the hydrogen transfer reaction family, BEP relations behave poorly for extremely exothermic and endothermic reactions. They proposed an alternative approximation, which is referred to as the Blowers–Masel approximation (BMA) in this study. In addition to coupling with LSRs for catalyst screening, BMAs can be applied to adjust the reaction barrier in other situations where the reaction enthalpy needs to be modified, such as thermodynamic sensitivity analysis,^17,18^ fitting thermodynamic data from experiments, and considering coverage effects in which the binding energy of an adsorbate changes with its coverage.^19−21^ Given their convenience and simplicity, BMAs could replace BEP relations for describing the activation energy as a function of the reaction enthalpy.

In this work, BMAs were implemented in Cantera^22^ and demonstrated on a study of catalytic methane partial oxidation (CMPO).^23^ Mazeau et al.^25^ investigated the best catalyst for CMPO^26^ by using LSRs to build microkinetic models on 81 hypothetical metals. In their work, the enthalpies of species on other metallic surfaces were scaled from a platinum surface using LSRs, but activation energies or reaction barriers were not changed. This work extends and builds on that study. To elucidate the use of BMAs in MKM, catalyst screening was conducted by applying LSRs to estimate species’ enthalpies on 81 hypothetical metals for the CMPO model both with and without BMAs to see the effect of scaling the activation energy. A CMPO model over platinum was made with a reaction mechanism generator (RMG),^27,28^ an open-source software for creating mean-field microkinetic models. A CMPO-BMA model was made by converting Arrhenius rate parameters in the CMPO model to BMA parameters while keeping the thermodynamic data fixed. The models with and without BMA parameters were then evaluated in a plug flow reactor (PFR) simulation with Cantera. Thermodynamic and kinetic sensitivity analyses were performed to compare the sensitivity of the reactions and species before and after the Arrhenius parameters were converted to BMA parameters. LSRs were then used to scale the CMPO and CMPO-BMA models to 81 hypothetical metal surfaces from which heat maps were generated to compare the descriptor values, such as CH_4_ conversion and full oxidation yield, and their first-order sensitivities. The two sets of heat maps were then compared to discuss the impact of using BMAs. The screening method presented in this article can serve as a starting point for further investigation, such as by performing DFT calculations on the identified metals.

## 2 Methods

### 2.1 Blowers–Masel Expression

Blowers and Masel^16^ have highlighted that the applicability of BEP relations becomes limited in cases of highly exothermic or endothermic reactions because it can lead to negative activation energies and poor estimates. They derive a new form of expression we term the Blowers-Masel approximation (BMA). The derivation follows from a few approximations, each of which they supported with detailed quantum chemistry calculations.^16,29,30^ First, consider an abstraction reaction of the form
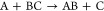
1

Blowers and Masel proposed that the potential energy surface *V*(*R*) for such a reaction can be described as
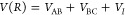
2where *V*_AB_ and *V*_BC_ are the potentials of AB and BC and *V*_I_ is an interaction potential. They describe *V*_AB_ and *V*_BC_ using Morse potentials and find that *V*_I_ can be described by
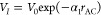
3where *V*_0_ and α_1_ are fitted parameters and *r*_AC_ is the distance between atoms A and C. With ABC colinear, the potential in (2) is rewritten as
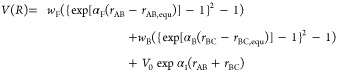
4where *w*_B_ is the B–C bond breaking energy, *w*_F_ is the A–B bond forming energy, *r*_AB,equ_ and *r*_BC,equ_ are the equilibrium bond lengths, and α_B_ and α_F_ are the force constants for the bonds. The saddle point is located by setting the derivatives of *V*(*R*) with respect to *r*_AB_ and *r*_BC_ to zero. By presuming a form of Badger’s rule where
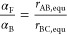
5and using *w*_0_ to represent the average of the bond breaking energy and the bond forming energy
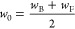
6the analytical expression of the BMA can be simplified to
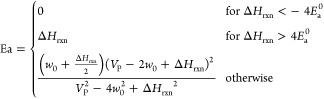
7where
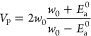
8

*E*_a_^0^ is the intrinsic activation energy and equals the activation energy when Δ*H*_rxn_ = 0, and *w*_0_ is a parameter with units of energy. In the derivation that applies to hydrogen transfer reactions it is the average of the bond dissociation energies of the bond being broken and the bond being formed. It was found that *w*_0_ does not significantly change the fitting results for the surface reactions.

Blowers and Masel^16^ demonstrated their expression fit well the data of the 151 hydrogen transfer reactions tabulated by NIST.^31^ Because the validation data were for gas-phase reactions, the expression was not previously shown to be applicable to surface reactions. To address this, we here use a variety of heterogeneous surface reactions, mostly taking *E*_a_ and Δ*H* data from Catalysis-Hub,^32^ an open DFT database for surface reactions.

**Figure 1:**
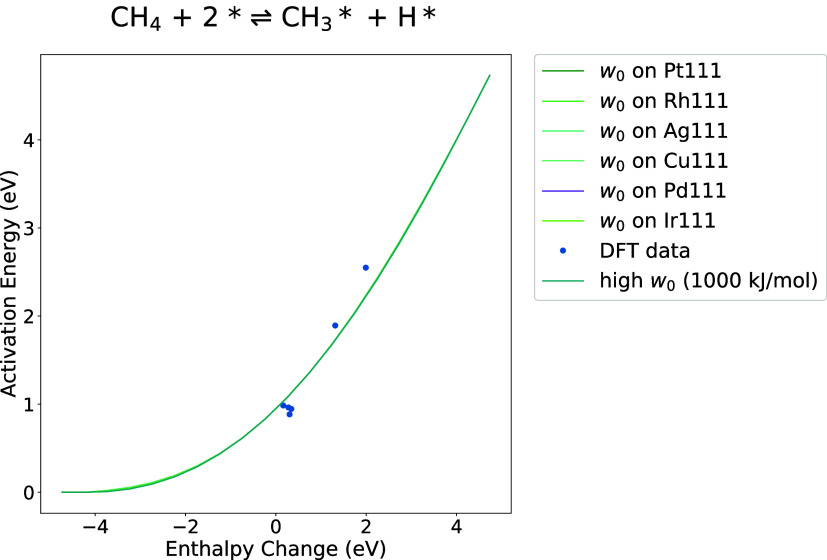
Comparison of BMA fitting with *w*_0_ on the 111 facet of different metals.

The weak influence of *w*_0_ in BMA fitting is demonstrated in Figure 1 using the reaction  where * represents the surface site.

The points show *E*_a_ and Δ*H* values from Catalysis-Hub for different (111) metal surfaces. The lines show a best-fit BMA curve using *w*_0_ values corresponding to each metal surface as well as to a very high *w*_0_ value of 1000 kJ/mol. The lines are coincident, showing that *w*_0_ has little effect on the BMA fitting outcomes as no prominent variation is observed. This holds as long as *w*_0_ > 2*E*_a_^0^, so an arbitrary high value can be used. Due to the insensitivity of BMA fitting to the value of *w*_0_ when it is high enough, we henceforth assign it a value of 1000 kJ/mol in the reaction rate calculation for convenience.

**Figure 2:**
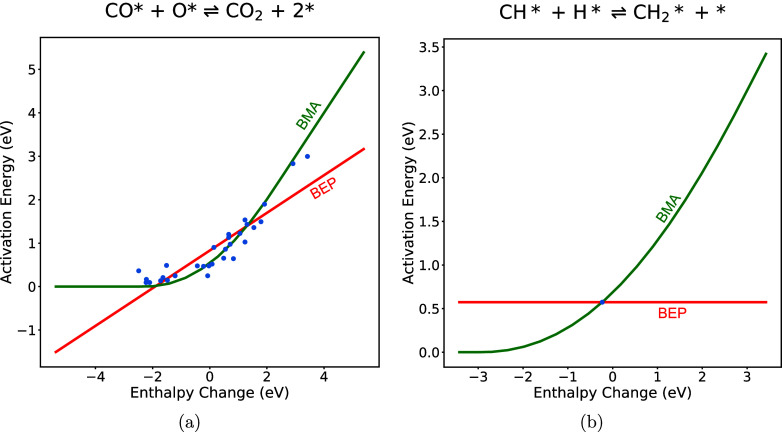
(a) Comparison of BMA and BEP fitting with *w*_0_ = 1000 kJ/mol.^33^ (b) Comparison of BMA and BEP fitting with only one DFT data point.^34^ BEP fitting is shown in red, BMA fitting is shown in green, and the DFT data are shown as blue dots.

Given the minimal influence of *w*_0_ on the activation energy, *E*_a_^0^ is the only parameter to be determined from the activation energy and enthalpy of a reaction. This dependence on a single parameter is a major benefit of the BMA approach. Therefore, any Arrhenius parameters of a reaction can be converted to BMA parameters with only knowledge of the enthalpy of the reaction. BMA parameters allow the activation energy to be scaled accordingly if the reaction enthalpy is changed by LSR or other causes. Following that, a comparison was performed between the BEP fittings and BMA fittings with *w*_0_ = 1000 kJ/mol in the BMA fitting. Figure 2a compares the two fittings for carbon monoxide oxidation reaction CO* + O*  CO_2_ + 2* on metal oxide surfaces, with the DFT data generated and compiled by Kropp and Mavrikakis.^33^ This reaction was chosen for demonstration because the available reaction enthalpy data cover a wide range from −4*E*_a_^0^ to 4*E*_a_^0^. The root mean squared error (RMSE) of BMA is 0.22, while the RMSE of BEP is 0.32. Note that the BEP fitting would predict negative activation energies at low Δ*H* values (<−1.9 eV) and barriers below the enthalpy of reaction for high Δ*H* > 1.4 eV, whereas the BMA predictions do not have this problem. The BMA expression also fits the DFT data better than the BEP expression does in these extremes and captures the curvature. Similar BEP and BMA comparisons were done for 11 reactions in the CMPO model that have multiple DFT data on Catalysis-Hub, and the RMSEs all show a high degree of similarity, as included in Supporting Information Figure S1.

Figure 2b is the comparison between BMA and BEP when there is only one set of enthalpy and activation energy data available for reaction CH(s) + H(s)  CH_2_(s). It is worth noting that a BMA fitting can still be generated, whereas a BEP fitting is a flat line with an unknown slope (here assumed to be 0). This derivation of a BMA expression from a single reaction rate is how the Arrhenius rates are converted to BMA rates, as shown in Section 2.2.

The BMA is implemented in Cantera as a rate type, which requires the pre-exponential factor *A* and accepts a temperature exponent *b*, like the modified Arrhenius equation in eq 9.
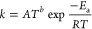
9

Instead of activation energy *E*_a_ in the Arrhenius rate, the BMA rate expression requires users to specify the intrinsic activation energy as *E*_a_^0^ and the average bond dissociation energy as *w*, as defined in eqs 7 and 8, while the calculation of reaction enthalpy is handled internally by Cantera. An example of BMA rate expression input is
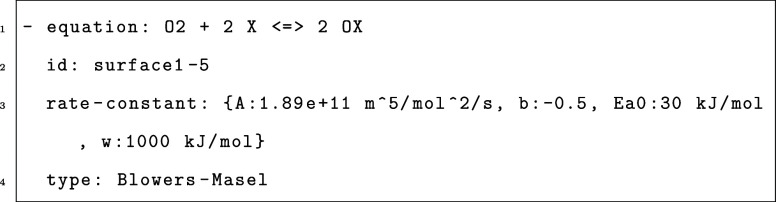


where the first line is the reaction equation and the second line represents the reaction index. The fourth line designates the reaction type, which Cantera reads to select the appropriate built-in function for estimating the rate coefficient. It is important to highlight that the BMA rate constant is evaluated on the basis of the enthalpy at the temperature in the current system, rather than at 298 K. While Cantera generally works internally in SI units, input values can be provided using many different units. The units can be specified using a “units” mapping in the Cantera YAML input file^35^ or written specifically for individual values like in this example. Modifications related to BMA were added to both C++ and Python code in Cantera to enable the flexibility of using the code in multiple languages for reactor simulations.

### 2.2 Model Generation

Reaction Mechanism Generator (RMG)^27,28^ is an open-source software to automatically build microkinetic models, with built-in thermodynamic and kinetic estimators and a database. RMG estimates the enthalpy of formation, entropy, and temperature-dependent heat capacity of a surface species by adding the properties of the gas-phase counterpart of the surface species and the difference caused by adsorption.^36^ LSRs^10^ are used to scale the binding energy of a surface species from Pt(111) to estimate adsorbate enthalpy on other metals. The adsorption estimates on Pt(111) are currently based on 69 species containing C/H/O/N. The data were calculated by Blondal et al.^37^ using the Vienna ab initio simulation package^38,39^ with the BEEF-vdW functional^40^ interfaced with Atomic Simulation Environment.^41^ The gas-phase thermodynamic properties are calculated using Benson’s group additivity and DFT.^27^

The reactions are determined by RMG kinetics families, which describe the bond connectivity changes from reactants to products. Each reaction family has a hierarchical tree for rate estimations. Once the reaction family is chosen, the associated tree is searched to match the species, and the reaction with the closest functional groups is used if there is no exact match. The rate parameters in the trees are either acquired from a published model or estimated by averaging the parameters of similar reactions in the same family.^28^ Besides the mentioned methods, published models are incorporated in RMG to provide thermodynamic and kinetic parameter tables, which are referred to as libraries. The parameters are taken from the libraries if an exact reaction or species is found. RMG uses a rate-based algorithm^42^ for model generation, which starts by reacting user-defined “core” species, with the product species being added to the “edge”. During simulations under the user-specified conditions of interest, if the rate of production of an edge species is higher than the user-specified threshold, then it will be added to the core. The process is repeated until all edge species have a rate of production lower than the threshold. The threshold is set by
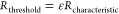
10where ε is a factor that can be assigned by user and the characteristic rate *R*_characteristic_ can be written as
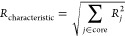
11where *R*_*j*_ is the rate of production of species *j* in the core.

To make the CMPO model in RMG, the methane partial oxidation models developed by Quiceno et al.^43^ and Mhadeshwar and Vlachos^44^ were chosen as the reaction libraries for modeling the surface reaction network, and the model developed by Burke and co-workers^45^ was used for gas-phase reactions. In order to provide the thermodynamics of the species, RMG relied on four libraries: **SurfaceThermoPt111**, **primaryThermoLibrary**, **thermo_DFT_CCSDTF12_BAC**, and **DFT_QCI_thermo**. These libraries contain thermodynamic data for gas and surface species obtained from ab initio calculations. **SurfaceThermoPt111** has the data for surface species on Pt, and the other three libraries have data for the gas-phase species. Species thermochemistry and reaction rates not found in these libraries are estimated by RMG. The model generation was started with 34 species, identified by Mazeau et al.,^25^ in the core, as shown in the RMG input file in the Supporting Information. Four surface batch reactors were added to verify the model for input ratios C/O = 0.6 and C/O = 2.6 at both 600 and 2000 K. The absolute and relative tolerances for the ODE solver in RMG were 1 × 10^–18^ and 1 × 10^–12^, respectively. The catalyst surface site density was set as 2.483 × 10^–9^ mol/cm^2^,^25^ and the remaining parameters can be found in the RMG input file in the Supporting Information. Nine carbon binding energies evenly distributed from −5.5 to −7.5 eV and 9 oxygen binding energies from −3.25 to −5.25 eV were combined to define 81 hypothetical metal surfaces. A separate RMG model was constructed for each hypothetical metal surface, and these individual models were then combined into a base model that included all possible species and pathways that can occur significantly on any of the 81 hypothetical metals.

The thermodynamic data of species on other metal surfaces were modified from platinum by RMG using LSR during the model generation,^25^ so the data were changed back to the original Pt(111) values when merging into the base model. Cantera was used to validate the base model. The BMA base model was made by converting all the Arrhenius parameters to BMA parameters using the enthalpy and activation energy of each reaction at 300 K and using the nonlinear equation solver in the SciPy package^46^ as described in Section 2.1. The BMA fitting results for each reaction can be found in the BMA Cantera input file in the Supporting Information. Two sets of models for 81 hypothetical metals were generated using LSRs to scale the species’ enthalpies from the original base model and the BMA base model. The models scaled from the BMA base model have different kinetics from the other set because the BMA changes the reaction barrier.

### 2.3 Reactor Simulation

Cantera was used to simulate the reactive flow through a PFR, which was represented as a chain of 7000 continuous stirred tank reactors (CSTRs), following the approach of Mazeau et al.^25^ This is sufficient to resolve the fast reactions in some of the simulations. The simulation results on platinum were compared with experimental data.^26^ The parameters of the reactor are shown in Table 1.

**Table 1:** PFR Parameters^26^ Used for Cantera Simulations

inlet gas temperature	800 K
reactor length	7 cm
reactor diameter	1.65 cm
catalyst porosity	0.81
catalyst area per volume	160 cm^–1^
inlet flow velocity	36.63 cm/s
catalyst length	1 cm
catalyst start position	1 cm

The composition of the inlet gas includes methane, oxygen, and argon, where the C/O ratios range from 0.6 to 1.4 incremented by 0.1 and from 1.6 to 2.6 incremented by 0.2. The ratio of Ar to O_2_ is 79: 21 at each C/O input ratio. The exit temperature, exit conversions of CH_4_ and O_2_, and the exit selectivities of CO, CO_2_, H_2_, and H_2_O at each C/O ratio were used as descriptor benchmarks to compare to experimental data.^26^

Methane conversion, synthesis gas yield, and full oxidation yield were used to measure model performance over all of the metals. Synthesis gas consists of CO and H_2_ and full oxidation refers to gas composed of CO_2_ and H_2_O. A value of 1 would be assigned to denote the complete synthesis gas yield or full oxidation of one gas species. However, both descriptors involve two gas products, so the values can be combined, allowing for a maximum possible value of 2.

### 2.4 Sensitivity Analyses

Kinetic and thermodynamic sensitivity analyses were performed on all models generated in this work to explore the influence of BMA expression on sensitivities. To calculate kinetic sensitivity, the rate of each surface reaction was perturbed by 1%, one at a time, and the change of the descriptor of interest at fixed position on the catalyst region was normalized by the change of the reaction rate, as written in eq 12
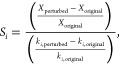
12where *S*_*i*_ is the kinetic sensitivity with respect to reaction *i*, *k*_*i*,original_ is the rate coefficient of reaction *i*, *k*_*i*,perturbed_ is the rate coefficient of reaction *i* after perturbation, *X*_original_ represents the value of a descriptor (e.g., CH_4_ conversion), and *X*_perturbed_ represents the descriptor value after the rate of reaction *i* is perturbed. The thermodynamic sensitivity was calculated by increasing the enthalpy of one adsorbate by 0.05 eV at a time and comparing the descriptor difference at a position on the catalyst. It is important to note that the enthalpy change is not modified proportionally (e.g., by 1%) because the definition of zero enthalpy is arbitrary. We perturb *H*_*j*_ rather than *G*_*j*_ because it is more straightforward in Cantera, but since Δ*G*° = Δ*H*° – *T*Δ*S*°, if we assume δ*S* = 0 then δ*G* = δ*H* anyway and our analysis is analogous to Campbell’s degree of thermodynamic rate control analysis.^47^

The expression of thermodynamic sensitivity can be written as eq 13
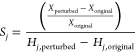
13where *S*_*j*_ is the thermodynamic sensitivity with respect to species *j* and *H*_*j*,original_ and *H*_*j*,perturbed_ are the enthalpies of adsorbate *j* before and after perturbation. The kinetic sensitivity is unitless, while the unit of thermodynamic sensitivity is eV^–1^.

Adding the BMA expression is expected to improve the accuracy of thermodynamic sensitivity analysis because the influence of enthalpies on reaction rates can be properly treated. In the limit of a small perturbation, the thermodynamic sensitivity in eq 13 can be written as
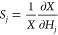
14where *X* is the descriptor value and *H*_*j*_ is the enthalpy of adsorbate *j*. Considering that *X* is calculated through a large set of ordinary differential equations or differential-algebraic equations, and the system includes the forward rate constants *k* and species enthalpies *H* as parameters, *X* in eq 14 can be expressed as *X*(*k*, *H*, ...). If *k*(*H*) is also a function of *H*, then using the chain rule, the partial derivative of *X* with respect to *H*_*j*_ is
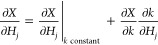
15

Here,  is the partial derivative of *X* with respect to *k* holding everything else constant,  is the derivative of *k* with respect to *H*_*j*_, and  represents the partial derivative of *X* with respect to *H*_*j*_ when treating *k* as a constant (i.e., ignoring the dependence of *k* on *H*_*j*_).

The Arrhenius rate expression is not affected by species enthalpy; therefore, the ∂*k*/∂*H*_*j*_ term of eq 15 is zero when using the original model with Arrhenius rates. On the contrary, reaction enthalpy is considered in the BMA rate expression, and because reaction enthalpy is a function of species enthalpy, the (∂*X*/∂*k*)(∂*k*/∂*H*_*j*_) term in eq 15 is included. Thus, models with BMA expressions will give more realistic thermodynamic sensitivity results than models with only Arrhenius expressions. It is worth noting that because all our models use reversible reactions, Cantera ensures thermodynamic consistency by deriving the equilibrium constant from Δ*G*_rxn_, and so the reverse rate coefficients depend on reaction enthalpy Δ*H*_rxn_ in both CMPO and CMPO-BMA models.

Positive sensitivity values indicate that increasing the reaction rate constant or species enthalpy leads to an increase in the descriptor values, while negative sensitivity values represent a decrease in the descriptor values. Reactions happen at an extremely small time scale, and species’ concentrations reach a steady state near the start of the catalyst zone in these simulations, so the catalyst surface to volume ratio is decreased to 5% of the value in Horn et al.^26^ when doing sensitivity analyses and descriptor screenings. As a consequence, the distance from the beginning of the reactor to reach the steady state is extended. This extension ensures that the chemistry happening at the descriptor sample position remains comparable between models with and without BMAs.

Calculating sensitivities by a finite difference method requires the comparison of small changes between numbers. When the numbers themselves are small (e.g., for hypothetical metals with a strong carbon binding energy that lead to almost no reaction), the comparisons become noisy and must be solved with very tight tolerances. The same hypothetical metals often have binding energies that lead to very stiff systems of ODEs, causing numerical difficulties to converge to tight tolerances. These issues do not plague the main results but make sensitivity analysis a challenge in some areas of the discovery space. To address the convergence issue and numerical noise for kinetic and thermodynamic sensitivity results, we averaged the results of multiple Cantera simulations with 6 varying error tolerances, using relative error tolerances (*r*_tol_) of 10^–*n*^ and absolute error tolerances (a_tol_) of 10^–2*n*^ for *n* = {5, 6, 7, 8, 9, 10}, at each C/O input ratio in the set {0.6, 1.0, 1.1, 1.2, 1.6, 2.0, 2.6}. This leads to 6 simulations for each species or reaction sensitivity calculation; simulations which failed as well as those positioned within the upper and lower quartiles were omitted from consideration, and the remaining results were averaged for analysis. As a result, the total number of simulations completed for thermodynamic and kinetic sensitivity analyses on 81 metals was 432,054.

## 3 Results and Discussion

### 3.1 BMA Expression Conversion

**Figure 3:**
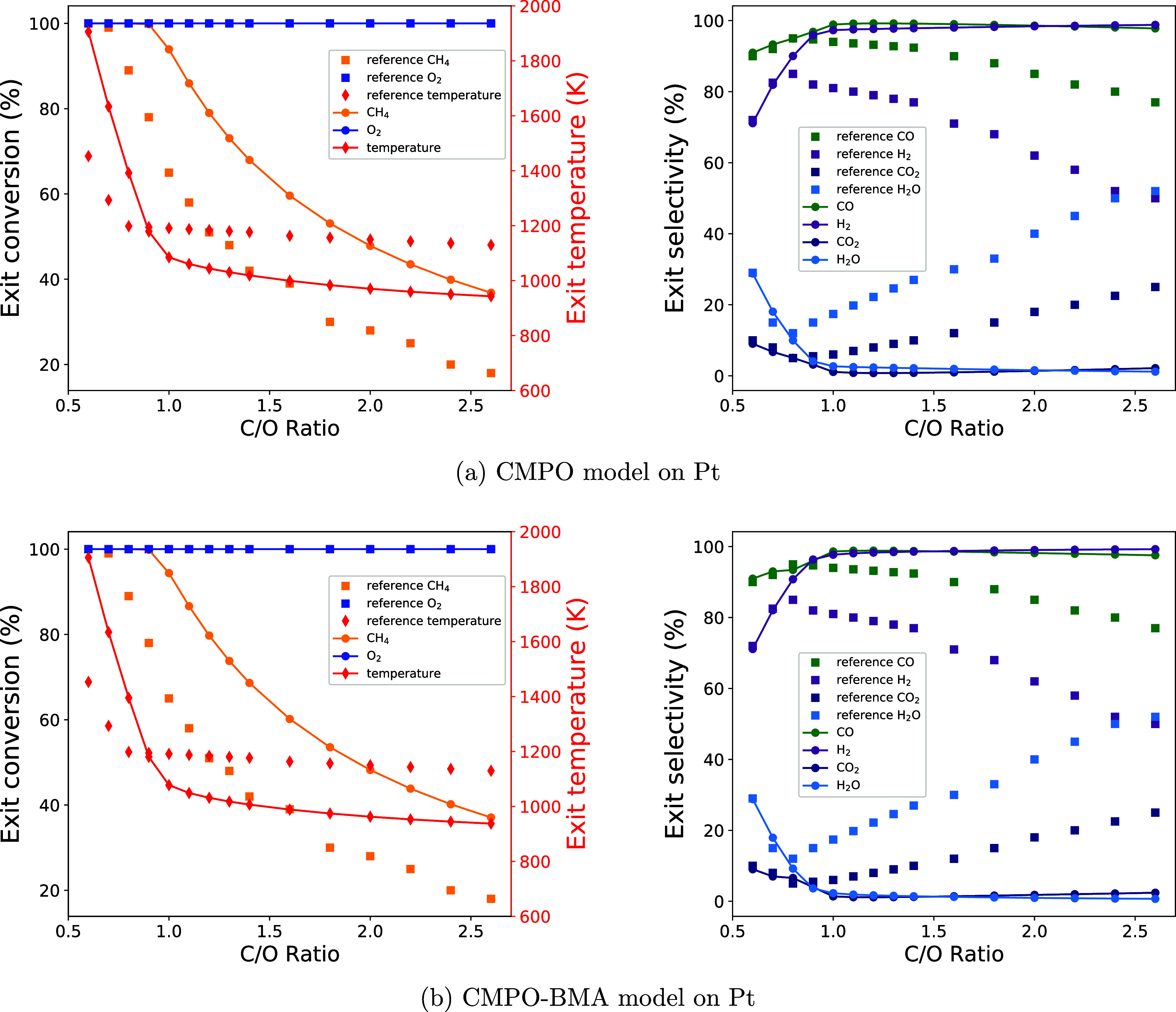
Simulation comparison between CMPO and CMPO-BMA models on Pt, the two plots in (a) are the species conversion and selectivity change with respect to C/O input ratio for CMPO model on Pt and the two plots in (b) are for the CMPO-BMA model on Pt. The square dots represent the reference (experimental) data from previous research,^26^ and the lines represent the simulation results.

The reaction pathways of the CMPO model can be found in the work done by Mazeau et al.^25^ The conversion of CH_4_ and O_2_, selectivity of synthesis gas (CO, H_2_) and full oxidized products (CO_2_, H_2_O), and temperature at the exit with respect to C/O input ratio are plotted against experimental data^26^ for the CMPO and CMPO-BMA models on Pt(111) in Figure 3.

The model was initially built and validated on rhodium,^25^ so there is a distinguishable disagreement with the experimental data on platinum. Given that the primary objective of this study is to explore the effect of BMA rates, the model is used as is. Despite the difference from the experimental data, the trends of descriptors for CMPO and CMPO-BMA base models are very close by comparing Figure 3a,b, which verifies a successful conversion from the Arrhenius rate to the BMA rate.

As implemented in Cantera, the activation energy in the BMA expression is calculated from the reaction enthalpy evaluated at the current system temperature, not a reference temperature of 298 K. The species’ enthalpies are calculated using NASA polynomials as a function of temperature. The activation energy in the BMA rate parameters is therefore slightly affected by the temperature, causing a small deviation from the Arrhenius rates. Thus, minor differences between the CMPO and CMPO-BMA models are observed in the synthesis gas conversion and full oxidation yield.

### 3.2 Sensitivity Analyses for CMPO and CMPO-BMA Models on Pt

#### 3.2.1 Kinetic Sensitivity on Pt

**Figure 4:**
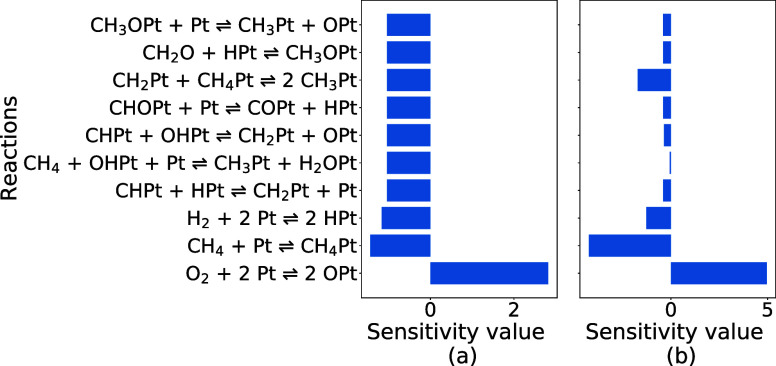
Kinetic sensitivity of CH_4_ conversion to reactions for CMPO (a) and CMPO-BMA (b) models on Pt(111). The top 10 sensitive reactions are drawn at C/O = 1.0.

As discussed in Section 2.4, kinetic sensitivity should remain the same for CMPO and CMPO-BMA base models because only the pre-exponential parameter is modified by 1%. As illustrated in Figure 4, the sensitivity of CH_4_ conversion at 1.045 cm (the 1045th CSTR in the simulation) to reactions with and without BMA rates is evaluated at C/O = 1.0, and the top 10 most sensitive reactions are plotted.

O_2_ + 2Pt  2OPt is the most positively sensitive reaction at C/O = 1.0 because the adsorbed oxygen further reacts with adsorbed carbon products to increase CH_4_ conversion. The CH_4_ physisorption reaction is the second most sensitive reaction, with a negatively sensitivity for CH_4_ conversion. Increasing the rate of CH_4_ physisorption reduces the coverage of atomic oxygen and atomic hydrogen, so the subsequent reactions are slowed down. The sensitivity analysis of the subsequent reactions, as depicted in Figure 4, reveals a considerable degree of similarity among them within the CMPO base model, which means that the CH_4_ conversion is equally sensitive to these reactions. In addition, the CMPO-BMA model has two reactions with high sensitivity, and the other eight reactions are much less sensitive. It is worth highlighting that both models agree on the top sensitive reactions, and the rank of most to least sensitive reactions does not change remarkably. The analogous ranks can be observed for the kinetic sensitivity of the synthesis gas and full oxidation yield in Figure S2. There are several factors that could cause the disagreement between the two models. The temperature exerts a subtle influence on the BMA rates, resulting in a slightly deviated reaction rate change in the CMPO-BMA. This variance, albeit small, can have a marginal impact on the ongoing reaction pathway within each CSTR. Consequently, the chosen position for the kinetic sensitivity analysis (the 1045th reactor) does not exhibit precisely identical conditions for the two models. Another likely reason is the numerical error caused by the solver. The sensitivity (eq 12) is calculated as the ratio of two small numbers, the numerator being the small difference between two much larger numbers (in this case, the conversion of CH_4_ in the 1045th CSTR). This amplifies any small discrepancies due to numerical imprecision within the tolerances of the solver.

Raising the C/O input ratio to 2.6 leads to the same trends, as shown in Supporting Information Figure S4. CH_4_ conversion is not sensitive to most reactions except the dissociative adsorptions of O_2_ and H_2_. This is primarily due to the fact that CH_4_ is the species with the majority of coverage on the surface at the higher C/O input ratio, and changing the rates of these two reactions enhances the coverage of adsorbed atomic oxygen or hydrogen, thus promoting subsequent reactions. Increasing the rate of O_2_ + 2*  2O* increases CH_4_ conversion, and increasing the rate of H_2_ + 2*  2H* decreases CH_4_ conversion in both base models. It is worth emphasizing that the kinetic sensitivity analyses for the CMPO and CMPO-BMA base models agree with each other for the most sensitive reactions, while showing differences in reactions that are relatively insensitive, and the sensitivity ranks of the two base models are more alike at higher C/O inlet ratio.

#### 3.2.2 Thermodynamic Sensitivity on Pt

**Figure 5:**
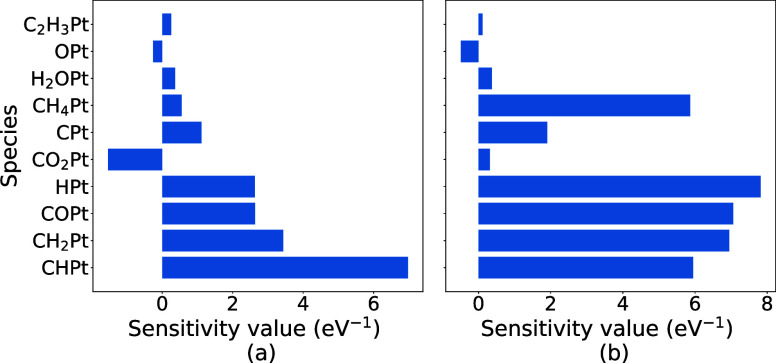
Thermodynamic sensitivity of CH_4_ conversion to species for CMPO (a) and CMPO-BMA (b) models. The top 10 sensitive reactions are drawn at C/O = 1.0.

Meanwhile, thermodynamic sensitivity analysis (Figure 5) shows that the sensitivity of CH_4_ conversion with respect to the enthalpy of each adsorbate is quite different between the models with and without BMA. Conversion is much more sensitive to changes in the enthalpy of CH_4_* with the base BMA-CMPO model than that with the base CMPO model at C/O = 1.0, and the most sensitive species is shifted from CH* to H* after the rate type is converted. In addition, increasing the enthalpy of adsorbed CO_2_* decreases the conversion of CH_4_ with Arrhenius rates, while it increases the conversion with BMA rates. The similar sensitivity value shifts can be seen for the synthesis gas yield and full oxidation thermodynamic sensitivity analysis, as shown in Figure S3. The thermodynamic sensitivity analysis shows that the Arrhenius to BMA rate type modification can change the sensitivity of descriptors to species’ enthalpies and could even lead to an opposite correlation between a descriptor and a species enthalpy. During the sensitivity analysis, the enthalpy of species was perturbed by 0.05 eV, subsequently impacting the enthalpy of reactions involving these species. Notably, in the CMPO-BMA model, the forward reaction barriers are enthalpy-dependent, while in the CMPO model, they remain unaffected. Thus, this difference led to a discrepancy in the thermodynamic sensitivity analyses, as shown in eq 15.

### 3.3 Descriptor Screening Results

**Figure 6:**
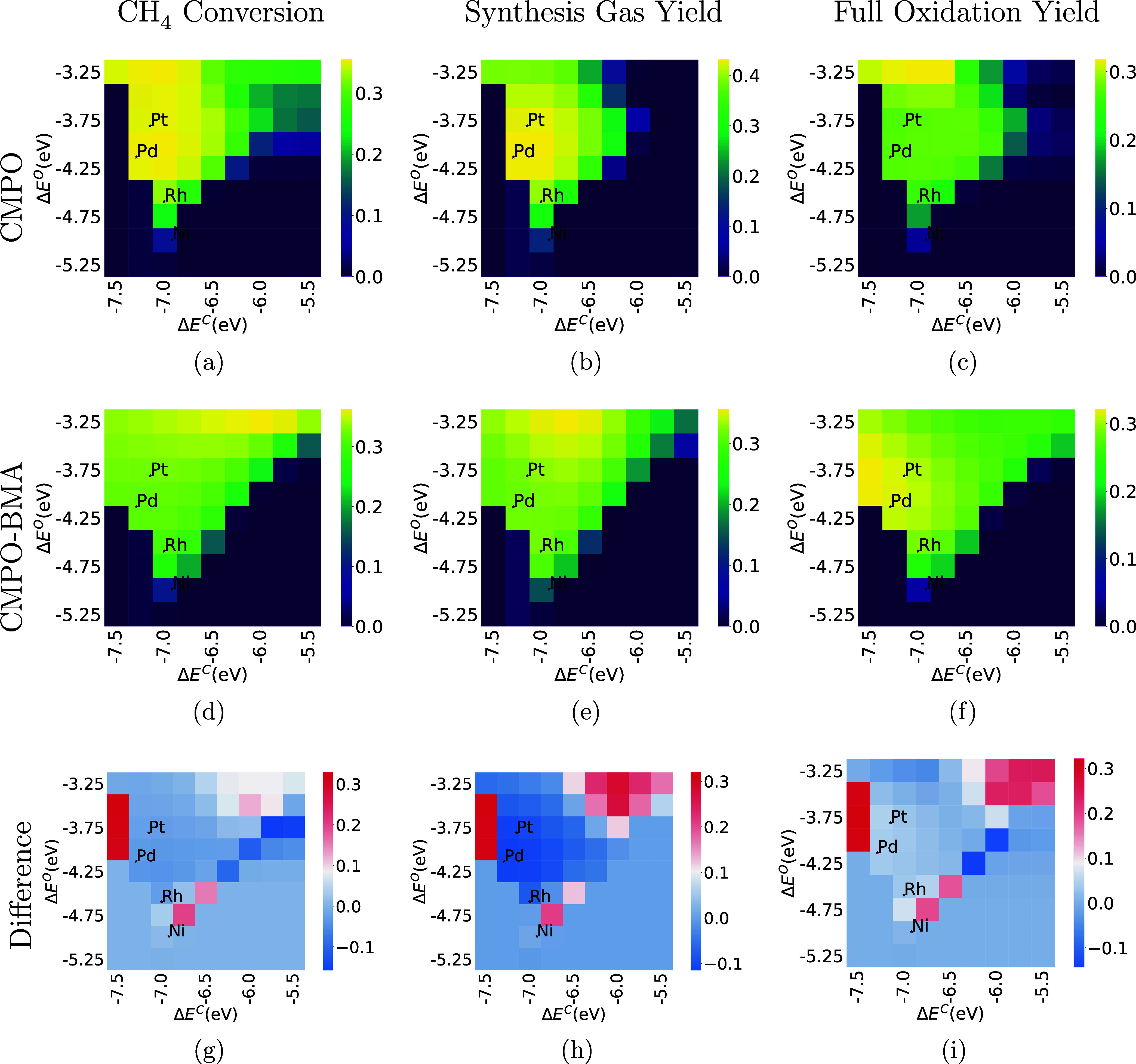
Comparison of CH_4_, synthesis gas, and full oxidation conversion at C/O = 0.6 between CMPO (a–c) and CMPO-BMA (d–f) models. The third row is the difference between CMPO-BMA and CMPO models (g–i). The *y*-axis represents the binding energy of atomic oxygen, the *x*-axis represents the binding energy of atomic carbon, and each pixel represents a hypothetical metal interface.

**Figure 7:**
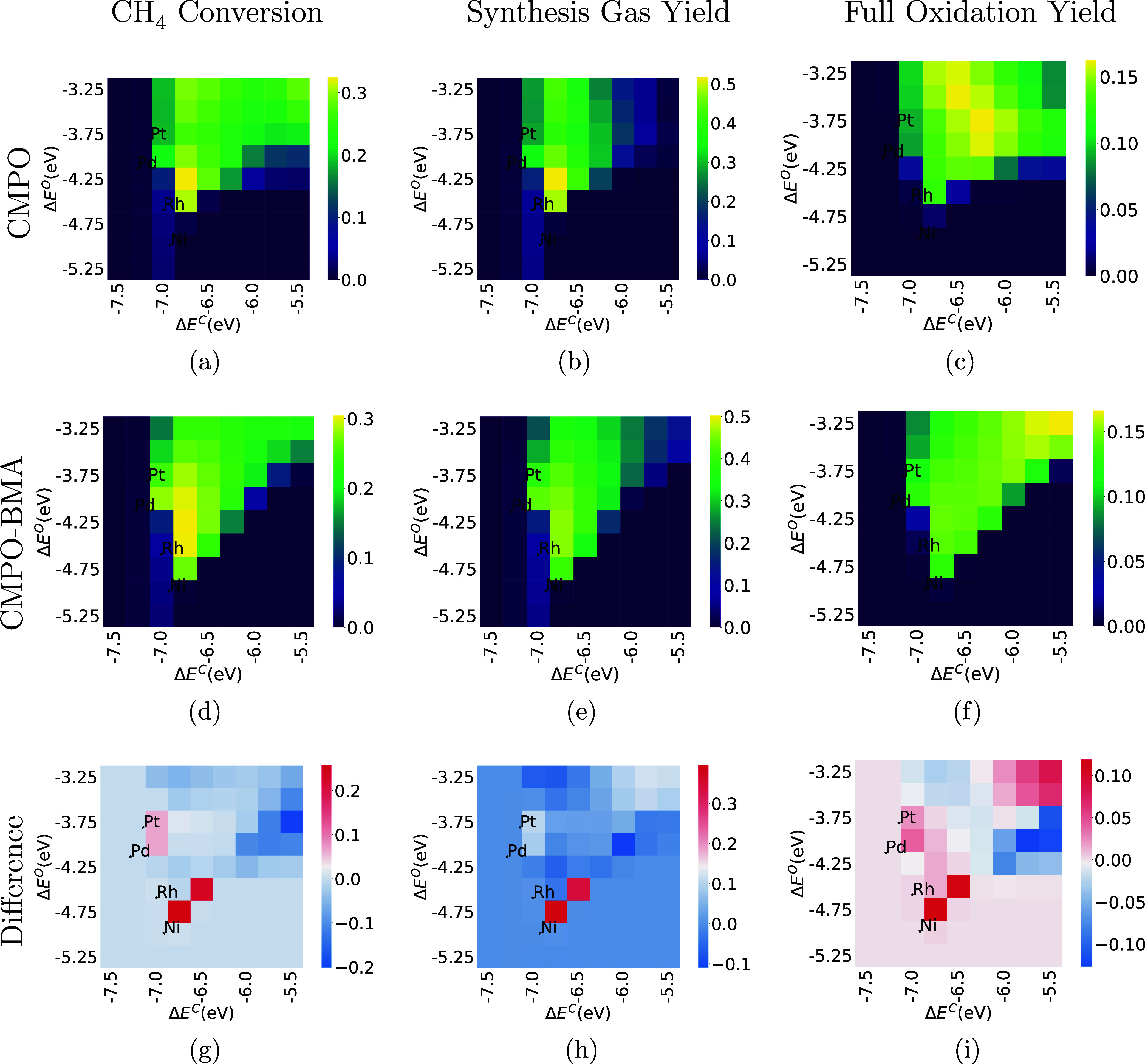
Comparison of CH_4_, synthesis gas, and full oxidation conversion at C/O = 1.0 between CMPO (a–c) and CMPO-BMA (d–f) models. The third row is the difference between CMPO-BMA and CMPO models (g–i). The *y*-axis represents the binding energy of atomic oxygen and the *x*-axis represents the binding energy of atomic carbon. Each pixel represents a hypothetical metal interface.

Simulations were repeated for the CMPO and CMPO-BMA models over all the 81 hypothetical metals, and the values of CH_4_ conversion, synthesis gas yield, and full oxidation yield at 1.045 cm in the PFR, at C/O = 0.6, C/O = 1.0, and C/O = 2.6 are demonstrated in heat maps in Figure 6, 7, and S5. The main point here is that when using the BMA, the “hot spots” on the heat maps (the peaks of the volcano plots) move and conclusions about what is the “best” candidate catalyst might change.

Comparing Figure 6a,d, the catalyst resulting in the highest CH_4_ conversion at this point in the reactor moves from near palladium and platinum to a weaker carbon binding (Δ*E*^C^ is less negative) and weaker oxygen binding (Δ*E*^O^ is less negative). Comparing Figure 6b and e, the peak in yield of synthesis gas has moved to weaker oxygen binding (Δ*E*^O^ = −3.25 eV). Comparing Figure 6c,f, the peak in yield of full oxidation products (CO_2_ and H_2_O) has moved from Δ*E*^C^ = −6.75 eV, Δ*E*^O^ = −3.25 eV, toward stronger binding metals like Pt and Pd.

The values are plotted at 1.045 cm (the catalyst zone starts at 1.00 cm) to highlight differences between simulations. For all cases, by the end of the PFR (7.0 cm), all simulations were either inert or had similar high conversion and yield values, as shown in Figure S6 in the Supporting Information.

To investigate the reason for the shift in peak CH_4_ conversion from the central to upper-right area, metals at the two different peaks (Δ*E*^C^ = – 6.0 eV, Δ*E*^O^ = – 3.25 eV and Δ*E*^C^ = – 7.25 eV, Δ*E*^O^ = – 4.25 eV) were compared for both CMPO and CMPO-BMA models. The reaction path diagrams showing cumulative flux are shown in the Supporting Information Figures S7 and S8.

For the CMPO-BMA model on the metal with (Δ*E*^C^ = – 6.0 eV, Δ*E*^O^ = – 3.25 eV) at C/O = 0.6 (the hot spot in Figure 6d), the CH_4_ conversion is higher because some is converted to C_2_H_6_ and C_2_H_4_ through gas phase reactions, pathways that are not active at that point in the CMPO model on the same metal (Figure S7). The gas phase rate constants were not changed; therefore, this is likely because the temperature is higher in the BMA model due to faster exothermic reactions upstream in the adiabatic reactor simulation. This made the CMPO-BMA model consume 30% more CH_4_ than the CMPO model on the same metal.

On the metal with (Δ*E*^C^ = – 7.25 eV, Δ*E*^O^ = – 4.25 eV) (the hot spot in Figure 6a), the main reaction pathways for the CMPO and CMPO-BMA models are similar as seen in Supporting Information Figure S8, but the CMPO model has 12% higher amount of CH_4_ reacted.

Overall, the CMPO-BMA screening plots at 1.045 cm from the beginning of PFRs have different shapes compared with CMPO plots. The principal cause is that BMA rates make the catalysis proceed faster in general; therefore, the reaction pathways on certain metals differ from CMPO to CMPO-BMA models.

After the input C/O ratio was increased to 1.0, a similar story emerges (Figure 7). The peak of the volcano plot (the hot spot in the heat map) does not move significantly for CH_4_ conversion or synthesis gas yield, but it moves toward more weakly binding metals for the full oxidation yield (the bright zone moves up and right from Figure 7c–f). This is mostly due to the reaction of CO* + O*  CO_2_ + 2* being faster in the CMPO-BMA models.

### 3.4 Energy Diagrams

To visualize the impact of BMA rates on reaction barriers, the energy diagrams of primary pathways on the metal characterized by Δ*E*^C^ = – 6.0 eV and Δ*E*^O^ = – 3.25 eV are compared for CMPO and CMPO-BMA models at C/O = 0.6. This analysis aims to explain the heightened reactivity for that metal in the CMPO-BMA models in Figure 6d, contrasting with the CMPO results in Figure 6a. The pathway flux diagrams on the metal can be found in Supporting Information Figure S7.

**Figure 8:**
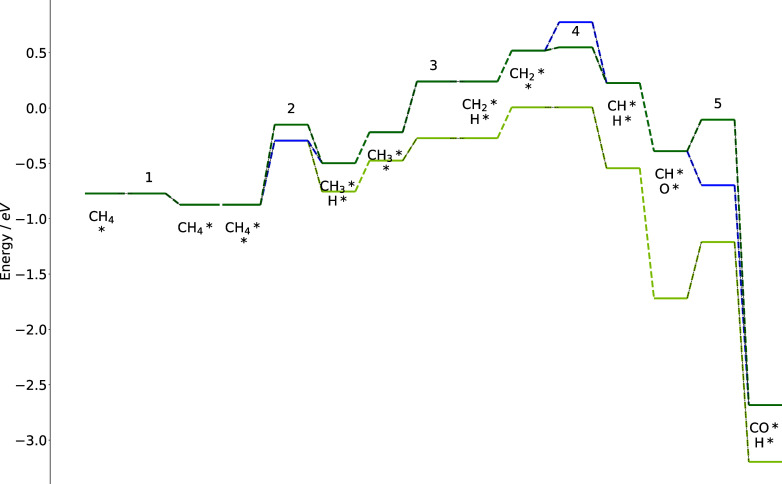
Energy diagrams for the main pathway on CMPO-BMA model on a hypothetical metal with Δ*E*^C^ = – 6.0 eV and Δ*E*^O^ = – 3.25 eV. The diagram comparison is drawn for CMPO model on Pt (yellow line), CMPO model (blue line), and CMPO-BMA model(green line).

Figure 8 explores the dominant reaction pathway for the CMPO-BMA model and the energy diagram for the identical pathway for the CMPO model. The reactions it goes through are listed in Table 2, and the Arrhenius and BMA rate parameters for steps 3, 4, and 5 are written in the reverse direction in Cantera input files. Some reactions in the middle are omitted from Figure 8 for simplicity.

**Table 2:** Dominant Reactions to Generate CO* for the CMPO-BMA Model on Metal with Δ*E*^C^ = – 6.0 eV and Δ*E*^O^ = – 3.25 eV

step #	Reaction
step 1	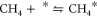
step 2	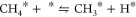
step 3	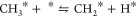
step 4	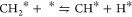
step 5	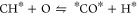

Gas-phase CH_4_ initially adsorbed on the surface (step 1) before reacting with a vacant site * to generate CH_3_* and H* (step 2). The third step happened through the reverse direction of the reaction given in the model as CH_2_* + H*  CH_3_* + *. The barrier was kept still by the BMA even though LSR made the reaction (as written) slightly more exothermic because the reaction enthalpy was smaller than −4*E*_a_^0^. The reaction to achieve the fourth step was also written in the reverse direction in the model as CH* + H*  CH_2_* + *. This reaction was less endothermic on the hypothetical metal than on Pt, so BMA lowered its barrier (green line) based on the expression for −4*E*_a_^0^ < Δ*H*_rxn_ < 4*E*_a_^0^. In contrast, the CMPO model did not lower the barrier (in the reverse direction) of the Arrhenius expression, leading to a higher barrier (blue line) for step 4 and a slower rate. Additionally, the activation energy in step 5 was estimated in the reverse, endothermic direction (from adsorbed CO* and H* to CH* and O*). LSRs altered it to be more endothermic on the hypothetical metal surface than on platinum. Thus, the activation energy is raised by BMA according to the expression for Δ*H*_rxn_ > 4*E*_a_^0^. The CMPO model (blue line) does not raise the barrier for step 5 despite the reaction becoming more endothermic in the direction written, leading to an unreasonably low submerged barrier, but the BMA model is able to adjust the barrier to a reasonable level (green line).

**Figure 9:**
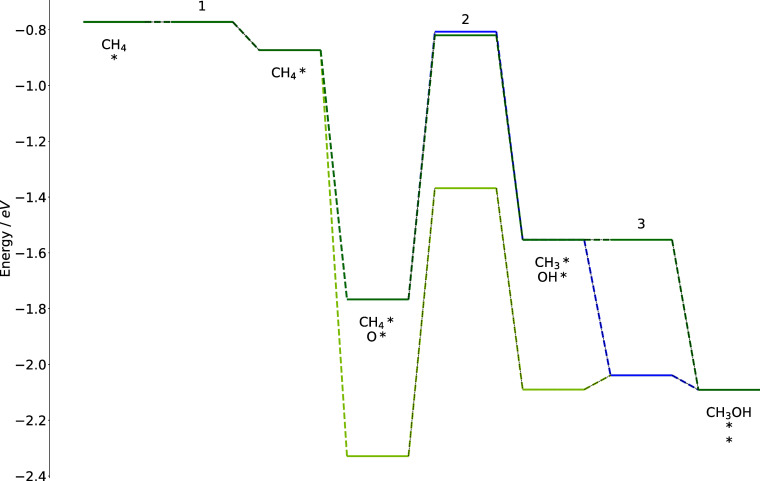
Energy diagrams for the main pathway on CMPO model on a hypothetical metal with Δ*E*^C^ = – 6.0 eV and Δ*E*^O^ = – 3.25 eV. The diagram comparison is drawn for the CMPO model on Pt (yellow line), CMPO model (blue line), and CMPO-BMA model (green line).

Table 3 shows the main pathway for the CMPO model on the same hypothetical metal, and the energy diagram is plotted in Figure 9.

**Table 3:** Dominant Reactions for the CMPO Model on Metal with Δ*E*^C^ = – 6.0 eV and Δ*E*^O^ = – 3.25 eV

step #	reaction
step 1	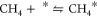
step 2	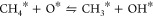
step 3	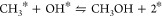

Step 1 is the same as the main pathway in the CMPO-BMA model in Table 2 and Figure 8. Then, CH_4_* reacted with O* to form CH_3_* and OH* (step 2) with a similar barrier in both models. Step 3, which produces gas-phase CH_3_OH, is presented as CH_3_OH + 2*  CH_3_* + OH* with a low barrier on Pt(111) estimated by RMG (yellow). When scaling to the metal with Δ*E*^C^ = – 6.0 eV and Δ*E*^O^ = – 3.25 eV, the dissociative adsorption reaction becomes more endothermic, but the Arrhenius CMPO model does not raise the barrier (blue), leading to a too-fast net rate of progress from CH_3_* to gas phase CH_3_OH, and this reaction thus replaced the reaction from CH_3_* to CH_2_* as the primary reaction path. The CMPO-BMA model, however, raises the barrier (green), slowing the formation of gas phase CH_3_OH, leaving more CH_3_* on the surface to eventually form CO.

In conclusion, the unrealistic rate of progress of the reaction CH_3_* + OH* → CH_3_OH + 2* was responsible for the low reactivity of the CMPO model on metal (Δ*E*^C^ = – 6.0 eV, Δ*E*^O^ = – 3.25 eV), and the BMA is able to raise the activation energy to prevent it. Consequently, a higher overall reactivity is seen on the same metal for the CMPO-BMA model.

### 3.5 Sensitivity Screening Results

Drawing upon previous investigations,^25^ the technique of kinetic sensitivity screening has been established to identify the reactions that govern the abrupt drops in conversions observed in the heat map, i.e., the cliff edges of the volcano. In addition, we performed the analysis of thermodynamic sensitivity to identify the species accountable for the heat map pattern. It is anticipated that the BMAs will induce alterations in the results of thermodynamic sensitivity as the intervention of BMAs, subsequent to species enthalpy modification through LSR, can potentially lead to shifts in reaction pathways. Therefore, the kinetic and thermodynamic sensitivity results for the CMPO and CMPO-BMA models are analyzed to explore the effect of BMA on the sensitivity screening results.

#### 3.5.1 Kinetic Sensitivity

**Figure 10:**
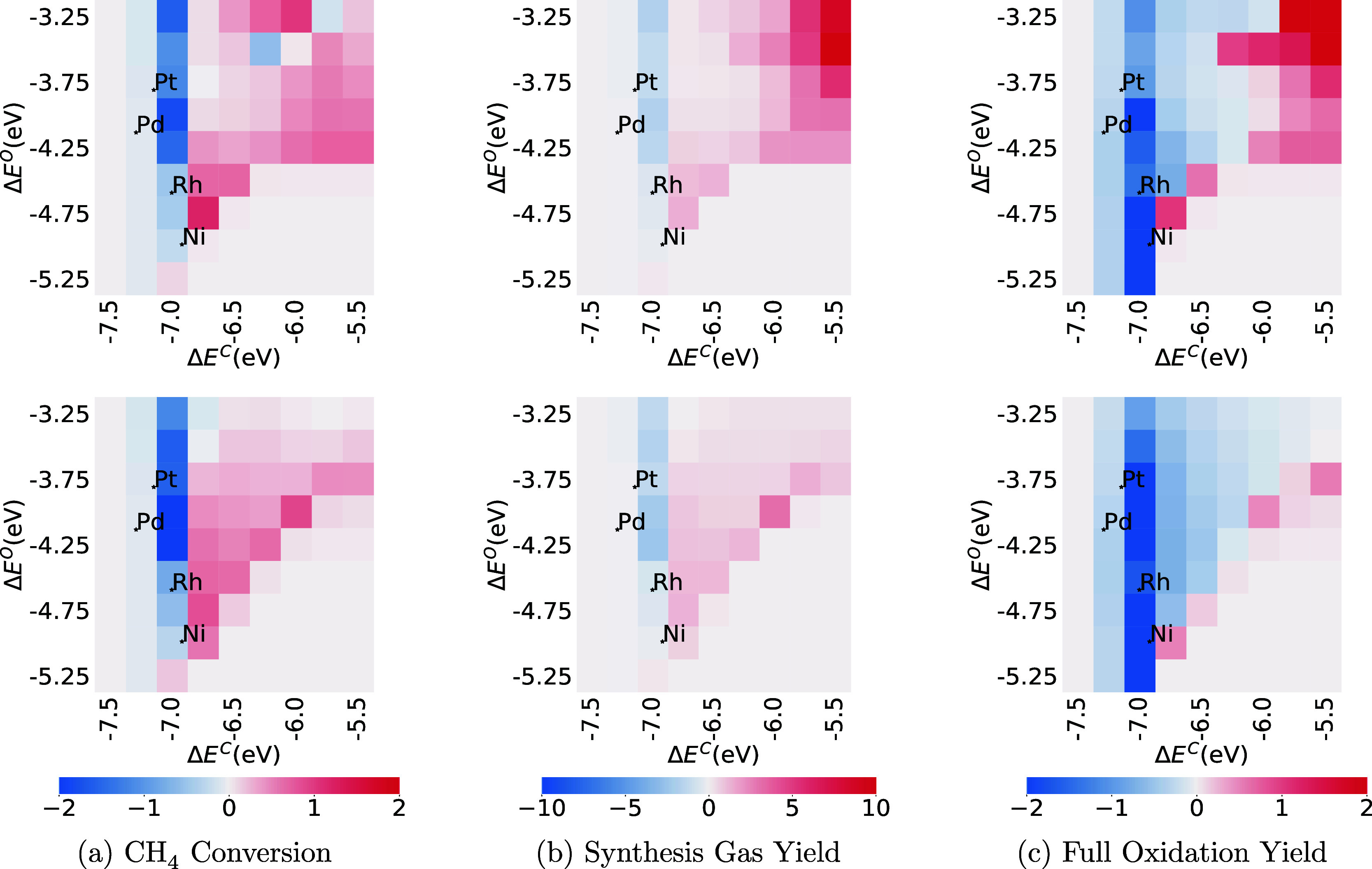
Kinetic sensitivity of CH_4_ conversion (a), synthesis gas (b), and full oxidation yields (c) to the rate of methane physisorption reaction at C/O = 1.0. CMPO models are on the top and CMPO-BMA models are at the bottom.

The kinetic sensitivity results on the base models identified that CH_4_ conversion is most sensitive to the adsorption of CH_4_ and O_2_, so the kinetic sensitivity screening encompassing CH_4_ conversion, synthesis gas, and full oxidation yield across the set of 81 metals was centered on these two reactions. As shown in Figure 10a, increasing the reaction rate of CH_4_ + *  CH_4_* causes an increase of CH_4_ conversion for both the CMPO and CMPO-BMA models on metals with carbon binding energy weaker than −6.75 eV. On the contrary, the metals on the left of the heat map, in the column where Δ*E*^C^ = – 7.0 eV, demonstrate strong negative sensitivity in all the maps in Figure 10. The surface-to-carbon bond grows stronger from the right to the left of the heat map, making it harder for carbonacious species to leave, leading to a higher coverage of carbonacious species. When the coverage is too high, the overall reactivity of the metal can be increased by slowing the adsorption reaction CH_4_ + *  CH_4_*) (negative sensitivity). When coverage is low, however, increasing the rate of adsorption increases the rate of reaction (positive sensitivity). Therefore, the pixels on the left of the heat map have reversed sensitivity compared to those on the right side. The same trends can be observed for synthesis gas and full oxidation yields as shown in Figure 10b,c.

**Figure 11:**
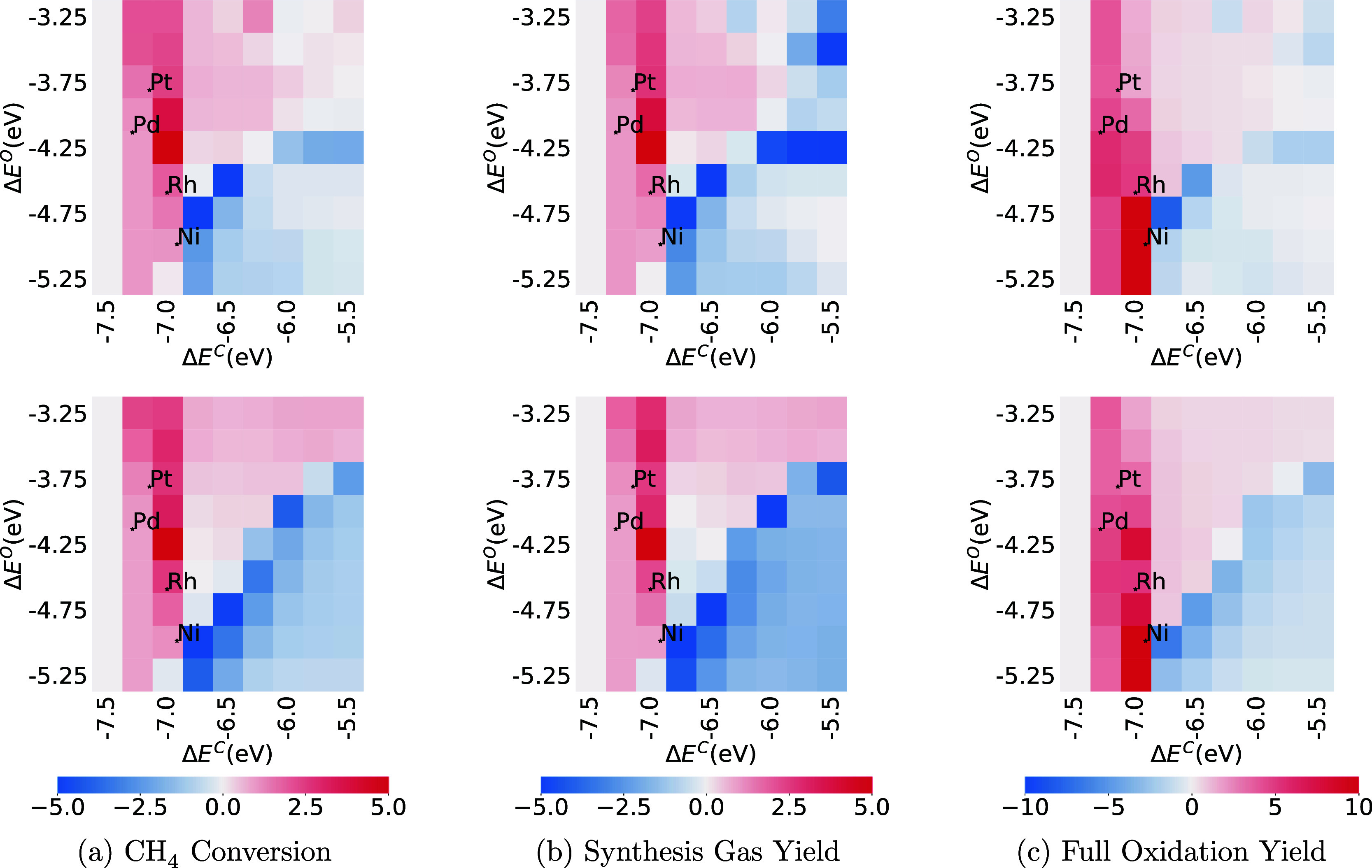
Kinetic sensitivity of CH_4_ conversion (a), synthesis gas (b), and full oxidation yields (c) to the rate of oxygen dissociation adsorption reaction at C/O = 1.0. CMPO models are on the top and CMPO-BMA models are at the bottom.

The sensitivity heat maps of dissociative adsorption of oxygen shown in Figure 11 have a reverse trend compared to the physisorption of CH_4_. The chemical process proceeds more rapidly due to 1% increase in oxygen adsorption rate on metals with strong carbon bonds (Δ*E*^C^ < – 7 eV) and weak oxygen bonds (Δ*E*^O^ > – 4 eV). This acceleration facilitates enhanced oxygen adsorption on the surface, fostering the further reactions. The negative sensitivities appear only on the metals with strong oxygen bonds and weak carbon bonds (bottom right corner), which have very low values in the descriptor screening maps in Figure 7, indicating that it is one of the reactions limiting the chemical process on these metals because the coverage of oxygen is too high.

Because the BMA changes the activation energy of reactions based upon reaction enthalpy, the CMPO-BMA reaction rates differ from the CMPO rates on most metals, so the kinetic sensitivity screening results have slightly different values but with similar trends. As discussed in Section 3.4, the rate discrepancies also changed the dominant reaction pathways, which explains the different shapes of the descriptor maps between the two types of model.

CH_4_ physisorption reaction and O_2_ dissociative adsorption are the most sensitive reactions, affecting most of the metal surfaces in the carbon and oxygen binding energy space explored, for both CMPO and CMPO-BMA models. Significant trends can be viewed across the metals, showing that these reactions are mostly responsible for the shapes of the descriptor heat maps. This suggests that incorporating BMA rates does not modify which reactions are the most sensitive reactions that dictate the shape of the descriptor heat maps.

#### 3.5.2 Thermodynamic Sensitivity

**Figure 12:**
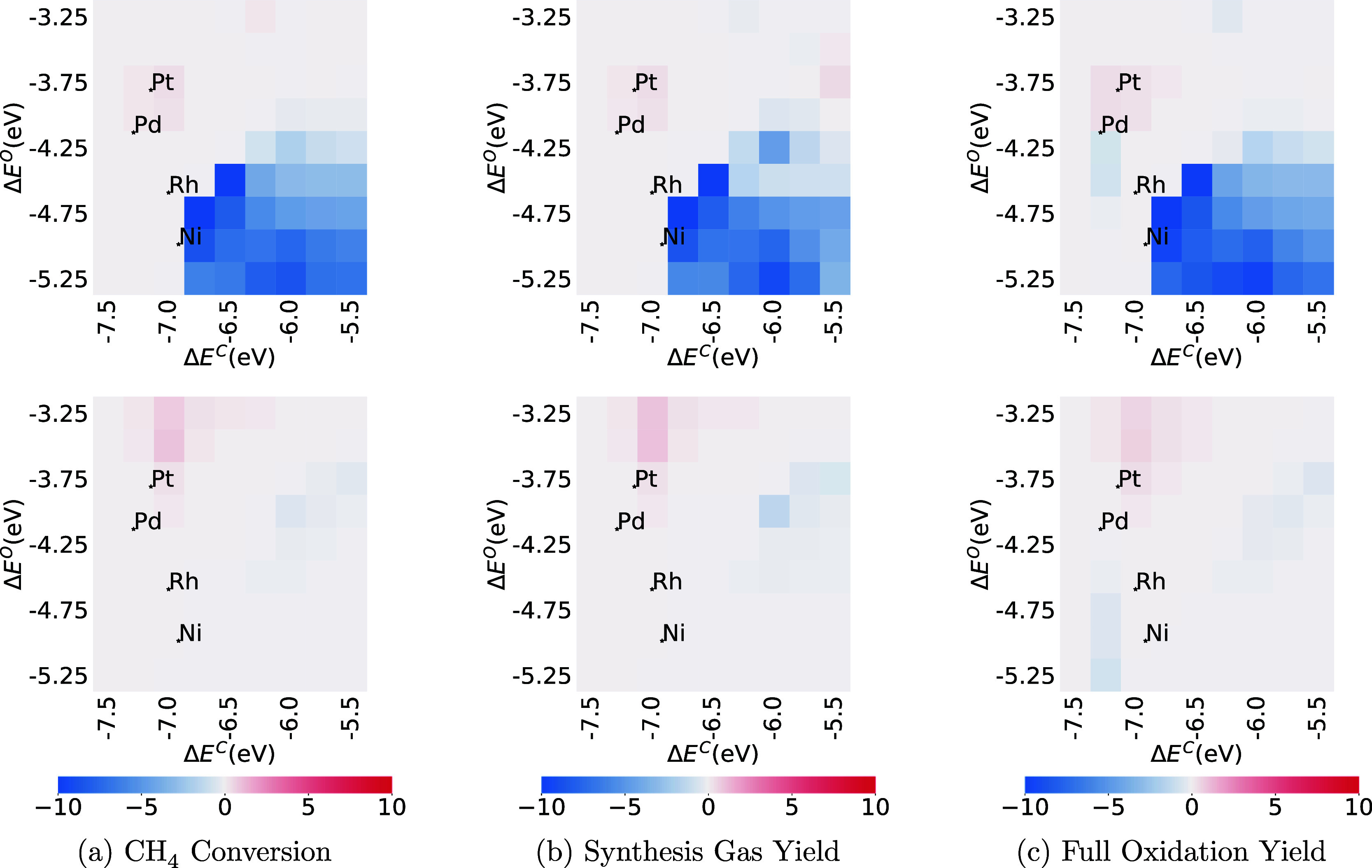
Thermodynamic sensitivity of CH_4_ conversion (a), synthesis gas yield (b), and full oxidation yield (c) to the enthalpy of adsorbed water (H_2_O*) at C/O = 1.0. CMPO models are on the top and CMPO-BMA models are at the bottom.

**Figure 13:**
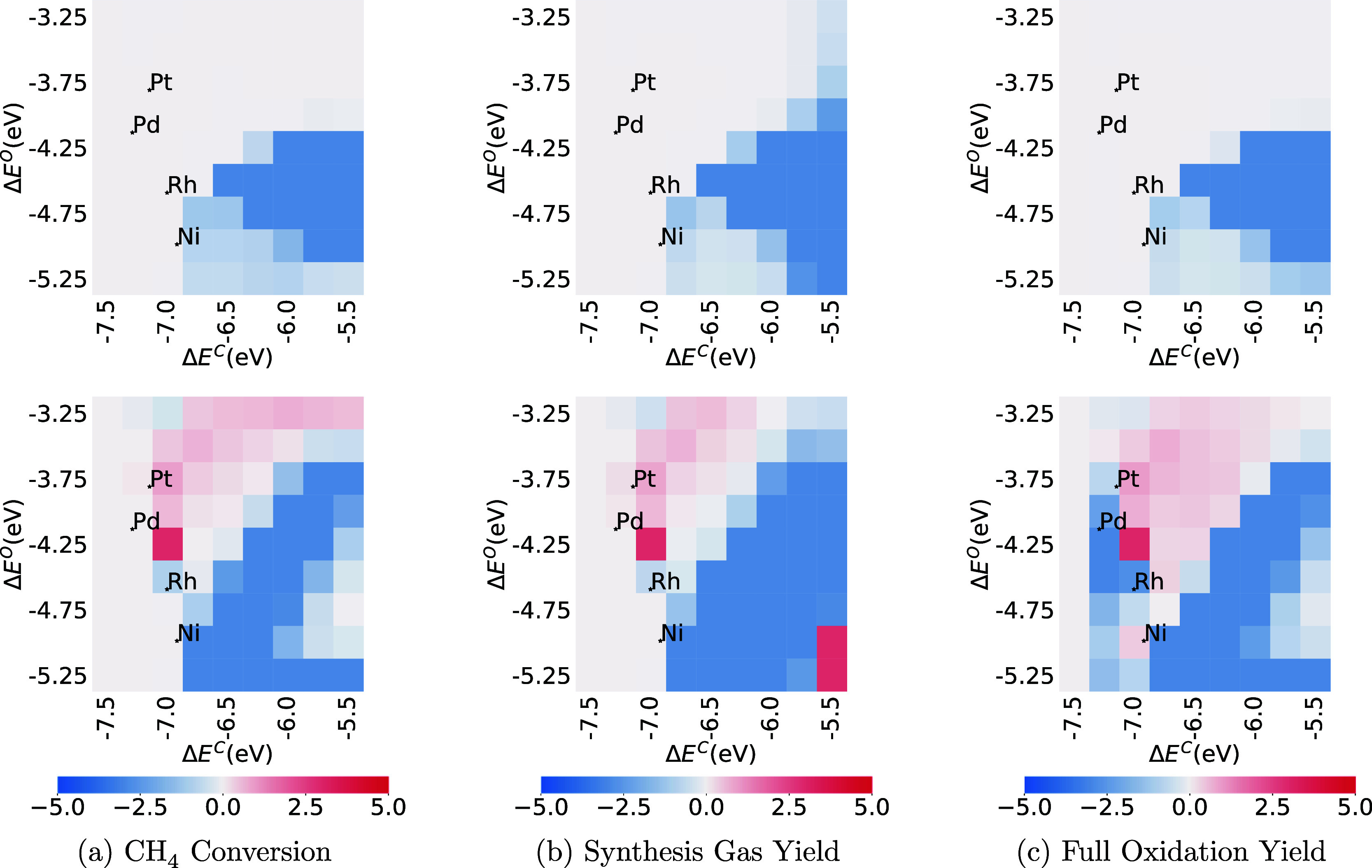
Thermodynamic sensitivity of CH_4_ conversion (a), synthesis gas yield (b), and full oxidation yield (c) to the enthalpy of adsorbed hydroxide at C/O = 1.0. CMPO models are on the top and CMPO-BMA models are at the bottom.

In contrast to the kinetic sensitivity, the thermodynamic sensitivity heat maps in Figure 12 and Figure 13 show that species’ thermodynamic sensitivity over the metals can change significantly after BMA rate substitution. Adsorbed water is negatively sensitive on about one-fourth of the metals, which have strong oxygen bonds and weak carbon bonds (bottom right) on the CMPO thermodynamic sensitivity heat maps of CH_4_ conversion, synthesis gas, and full oxidation yields in Figure 12. It suggests that the enthalpy of adsorbed water is one of the factors limiting the descriptor values. However, the sensitivity values on CMPO-BMA screening results in Figure 12 are more than 10 times smaller in general compared to CMPO models, indicating that the enthalpy of adsorbed water does not contribute as substantially to the descriptor values.

The disparity is also evident in Figure 13, where the sensitivity of CH_4_ conversion, synthesis gas, and full oxidation yield to the enthalpy of adsorbed hydroxide (OH*) exhibit pronounced negative values. While this effect is limited to metals in the lower right portion of the heat maps for CMPO models, a wider range of metals displays negative sensitivity values for CMPO-BMA models. It is noteworthy to highlight that for the CMPO-BMA models, on metals located in the active area (upper central portion) of the heat maps, the descriptors exhibit positive sensitivity (mostly with sensitivity less than 1 eV^–1^, except for the metal at Δ*E*^C^ = – 7 eV, Δ*E*^O^ = – 4.25 eV), with respect to the enthalpy of adsorbed hydroxide. However, in the corresponding areas of the CMPO model heat maps, these sensitivity values are consistently zero.

Despite the outliers caused by the solver imprecision at the bottom right corner of the thermodynamic sensitivity screening for synthesis yield for CMPO-BMA models in Figure 13b, the screening results of thermodynamic sensitivity of adsorbed hydroxide show that using BMA rates makes the species influential to methane oxidation on more metals compared to models with Arrhenius rates.

The thermodynamic sensitivity heat map analysis validates that BMA rates exert a considerable impact on the thermodynamic sensitivities of some species engaged in the chemical process. The introduction of BMA rates results in a shift in the dominant surface species governing the progression of methane oxidation across 81 metals. This influence stems from alterations in species enthalpy, which in turn affects the enthalpies and equilibrium constants of reactions involving those species. Consequently, the calculations of reverse reaction rates, relying on the equilibrium constants and forward rate constants, are perturbed. Unlike Arrhenius rates, which remain unchanged, BMA rates adjust the forward rate constants, thereby impacting the overall rates of the associated reactions. This adjustment results in distinctive variations in CH_4_ conversion, synthesis gas production, and full oxidation yields. In summary, applying BMA rates yields an alternative perspective on which species demand greater consideration during optimization or catalyst design.

### 3.6 Conclusions

The BMA rate expression was successfully implemented in Cantera,^22^ and CMPO models with and without BMA rates were compared. DFT data for 11 reactions in the CMPO base model were extracted from CatHub to validate the application of BMA to surface reactions. The CMPO base model on Pt was generated by using RMG, and the BMA rates were fitted on the basis of Arrhenius rates to make a CMPO-BMA base model. A catalyst screening analysis on 81 hypothetical metal surfaces was carried out in Cantera using both CMPO and CMPO-BMA models to investigate the influence of BMA rates. The hypothetical metals were characterized using a combination of carbon binding energies of −7.5 to −5.5 eV and oxygen binding energies of −5.25 to −3.25 eV.

Simulations for the CMPO and CMPO-BMA base models on platinum were carried out in a PFR, which was approximated as a series of CSTRs in Cantera, with C/O input gas ratios from 0.6 to 2.6 to replicate the experimental work.^26^ The models showed a noticeable difference compared with the experimental data, but general descriptor trends agreed. As the goal of this work was to explore the influence of BMA rates, we determined that the RMG model has good enough agreement with experimental results and would be used as a base model for comparison. The base model species concentration changes over the PFR were comparable, and the primary sensitive reactions for CH_4_ conversion remained unchanged after converting from Arrhenius to BMA rates. The thermodynamic sensitivity analysis for the base models with and without BMA rates revealed that the BMA rates result in a significant change (up to 4 times) in the sensitivity of CH_4_ conversion to species’ enthalpies.

The screening results illustrate that when using the BMA, the “hot spots” on the heat maps (the peaks of the volcano plots) move, and the “best” candidate catalyst selected by the analysis can be altered. The metals that are most effective for synthesis gas yield for CMPO models have stronger oxygen bonds (more negative binding energy) compared to the most effective metals for CMPO-BMA models at C/O = 0.6. Furthermore, the metals that attain the highest CH_4_ conversion and synthesis gas yield vary between the CMPO and CMPO-BMA models at a low C/O input ratio, while at high C/O input ratios, active metals and the descriptor screening heat maps exhibit similar patterns. The difference in the descriptor screening results between CMPO and CMPO-BMA models primarily arises from certain expedited chemical processes due to the BMA rates.

The kinetic sensitivity heat map helps identify which reactions are most responsible for the shapes of the descriptor heat maps. The descriptors are most sensitive to the CH_4_ adsorption and the O_2_ dissociative adsorption reactions with and without BMA rates. This leads to the conclusion that BMA rates do not affect the most sensitive reactions identified using kinetic sensitivity analysis. However, the thermodynamic sensitivity heat maps showed that adsorbed water is a rate-determining species for many CMPO models, while it is not for CMPO-BMA models over the metals screened. The thermodynamic sensitivity heat maps of adsorbed hydroxide (OH*), however, showed the opposite. This observation suggests that the use of BMA rates can alter the conclusions drawn from a thermodynamic sensitivity analysis.

This work added a new feature to the open-source simulation software Cantera, allowing reaction kinetics to be specified by using the BMA, in which the reaction barriers are a function of the reaction enthalpy. Unlike the BEP expression, this BMA form only requires one parameter (so it can be derived from a single reaction rate expression) and gives reasonable values when extrapolated to very high and low reaction enthalpies. We have shown that using BMA instead of simple Arrhenius expressions (with a fixed forward reaction barrier) during model analysis can lead to different results, both in the binding energies of the optimal catalyst and in the relative importance of specific adsorbate enthalpies. Incorporating the BMA rate description into Cantera enables a new workflow, demonstrated herein, allowing rapid screening of catalysts using linear scaling relationships (LSRs) and BMA kinetics within the simulation software with a single model input file. This can provide a starting point for a model of interest for further improvements. The workflow is not limited by the use of LSRs since the BMA kinetics could equally well be combined with modern machine-learned predictors of adsorbate energies. This could be an efficient first step in a catalyst screening investigation before further investigation (e.g., with DFT and then experiments) of any identified candidate catalysts.

## Supporting Information Available

BMA fittings for the DFT data extracted from Catalysis-Hub; kinetic and thermodynamic sensitivity of synthesis gas yield, and full oxidation for the CMPO and CMPO-BMA base Pt(111) models at C/O = 1.0; kinetic sensitivity of CH_4_ conversion, synthesis gas yield, and full oxidation for CMPO and CMPO-BMA base models at C/O = 2.6; descriptor screening results for CMPO and CMPO-BMA models at C/O = 2.6; descriptor screening results at the end of PFR for CMPO and CMPO-BMA models at C/O = 0.6; the reaction pathway for the CMPO and the CMPO-BMA model on the metal at (Δ*E*^O^ = – 3.25 eV, Δ*E*^C^ = – 6.0 eV); and the reaction pathway for the CMPO and the CMPO-BMA model on the metal at (Δ*E*^O^ = – 4.25 eV, Δ*E*^C^ = – 7.25 eV) (PDF)
RMG input file for the base model, microkinetic model for Pt(111) in Cantera format, microkinetic models on all the hypothetical metal surfaces with and without BMA rates, Python scripts and Jupyter notebooks for running the simulations and sensitivity analyses, fitting Blowers–Masel rates, and reproducing the plots in the paper. (ZIP) More scripts can be found on the Github repository https://github.com/comocheng/bm_project (ZIP)
