# Supplementary material for: Implementing the Blowers–Masel Approximation to Scale Activation Energy Based on Reaction Enthalpy in Mean-Field Microkinetic Modeling for Catalytic Methane Partial Oxidation
Source: ACS Catal. 2024 May 9;14(10):8013–29. doi: 10.1021/acscatal.3c05436 (PMC11106751; doi:10.1021/acscatal.3c05436)
Supplement: Supplementary file 1 — cs3c05436_si_001.pdf [file cs3c05436_si_001.pdf]

Supporting Information

Implementing Blowers-Masel Approximation to Scale Activation Energy Based on Reaction Enthalpy in Mean-field Micro-kinetic Modeling for Catalytic Methane Partial Oxidation

Chao Xu[a], Emily Mazeau[b], Richard H. West[a]

[a] *Department of Chemical Engineering, Northeastern University, Boston, MA, USA*
[b] *Oak Ridge National Laboratory, Oak Ridge, TN 37830, USA*

Email address: r.west@northeastern.edu (Richard H. West)

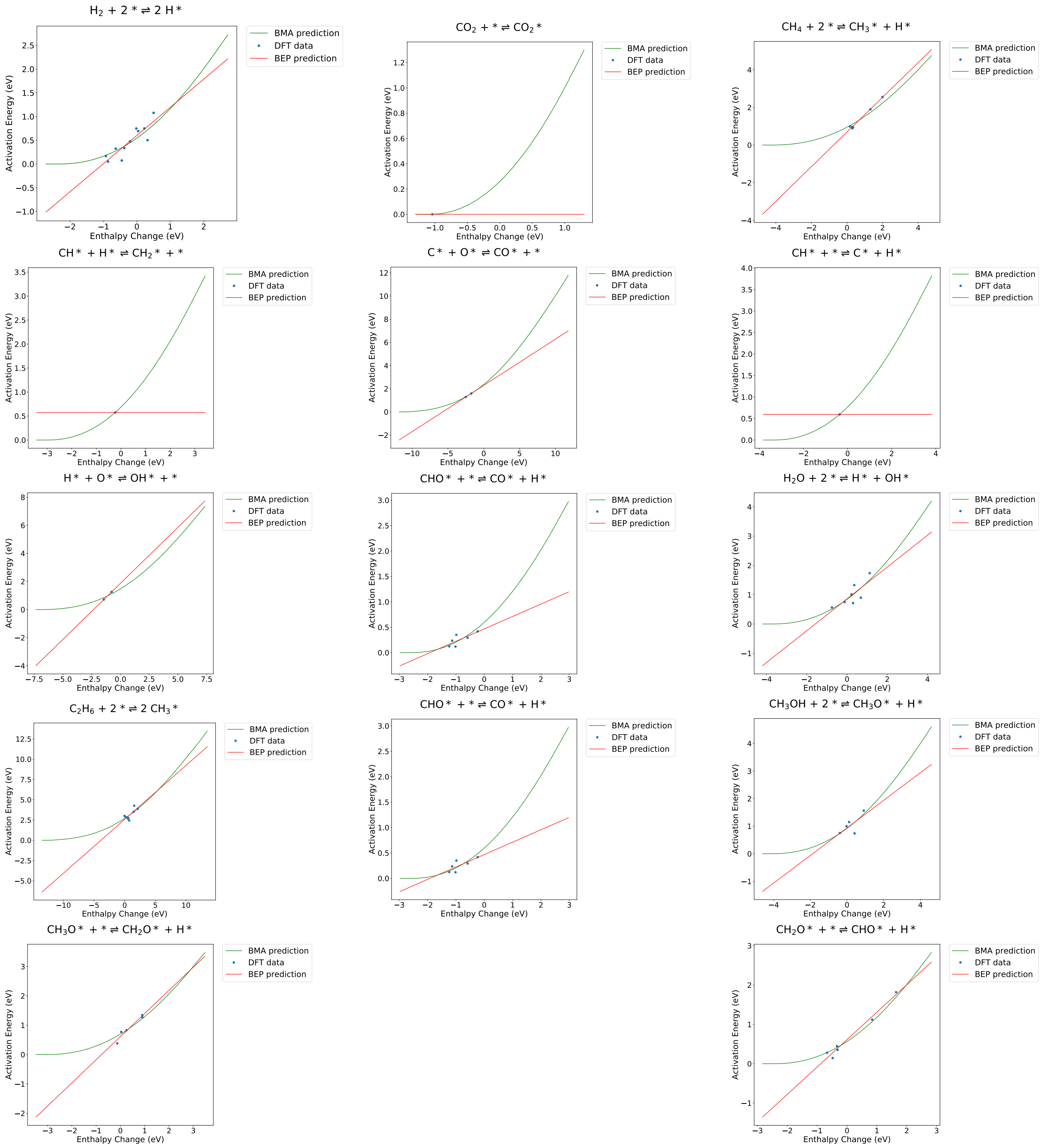

Figure S1: BMA fittings for the data extracted from Catalysis-Hub

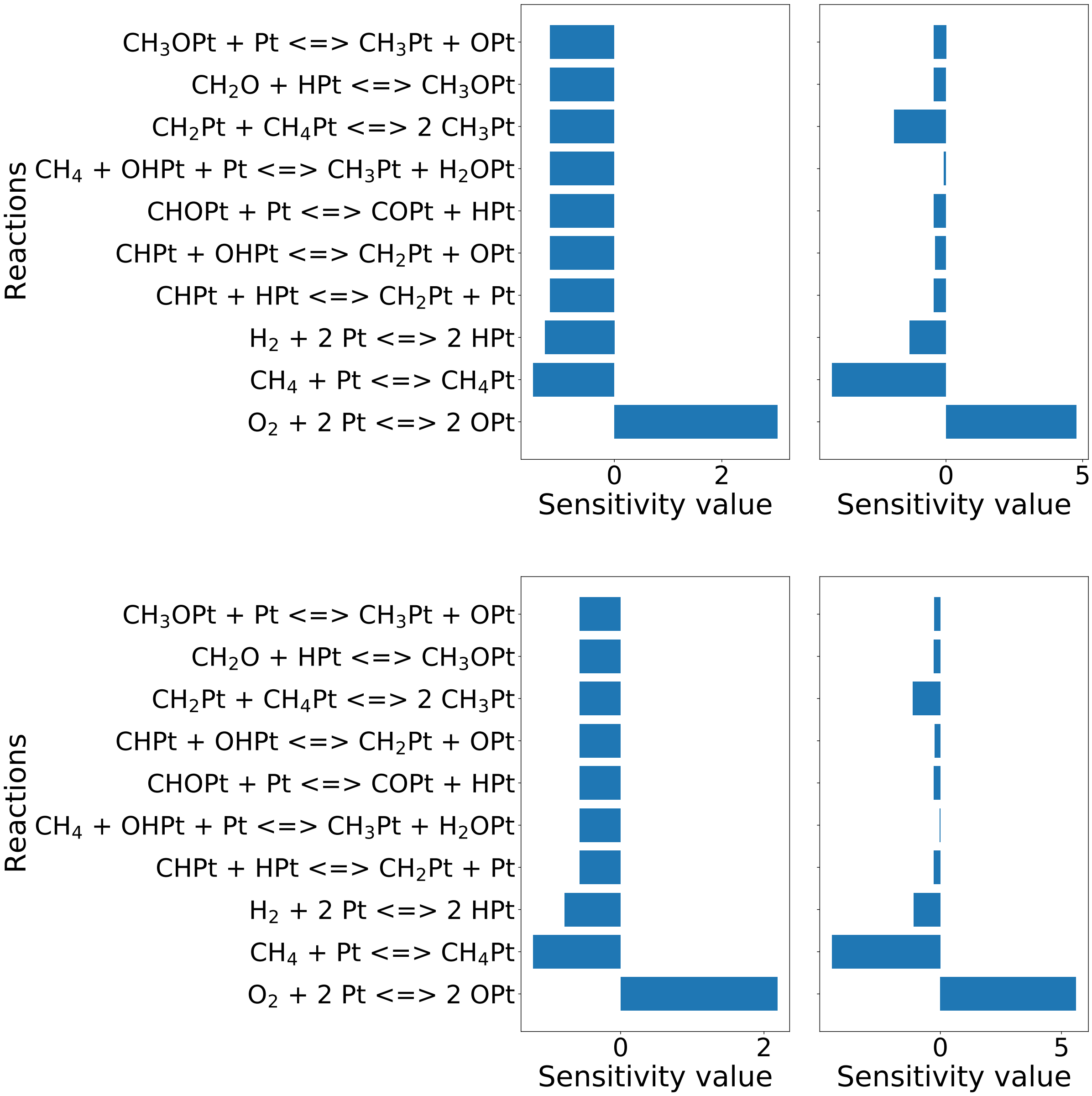

Figure S2: Kinetic sensitivity of synthesis gas yield (top plot) and full oxidation (bottom plot) comparison for the CMPO (left) and CMPO-BMA (right) base Pt(111) models at C/O=1.0

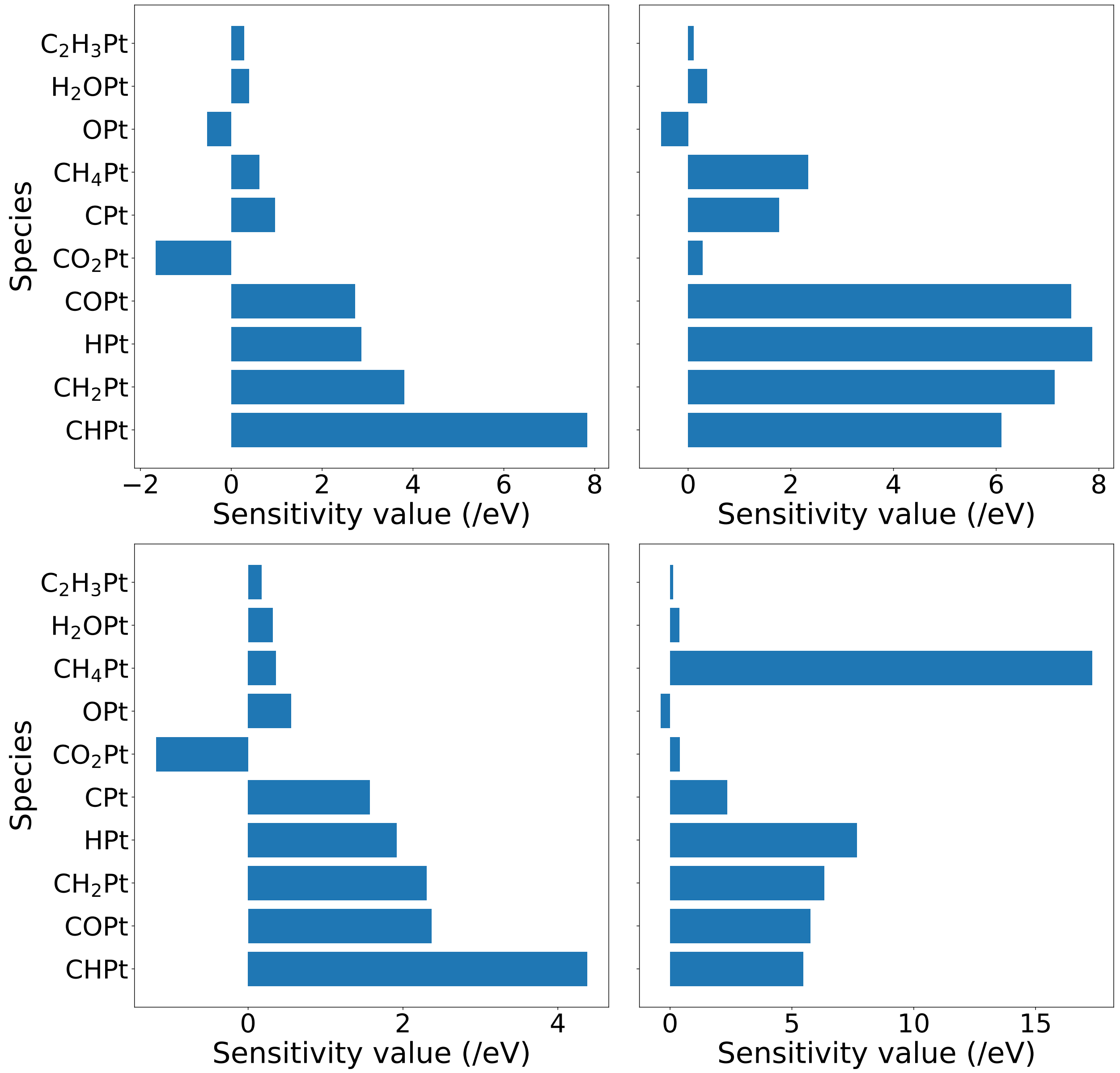

Figure S3: Thermodynamic sensitivity of sythesis gas conversion (top plot) and full oxidation (bottom plot) comparison for the CMPO (left) and CMPO-BMA (right) base Pt(111) models at C/O=1.0

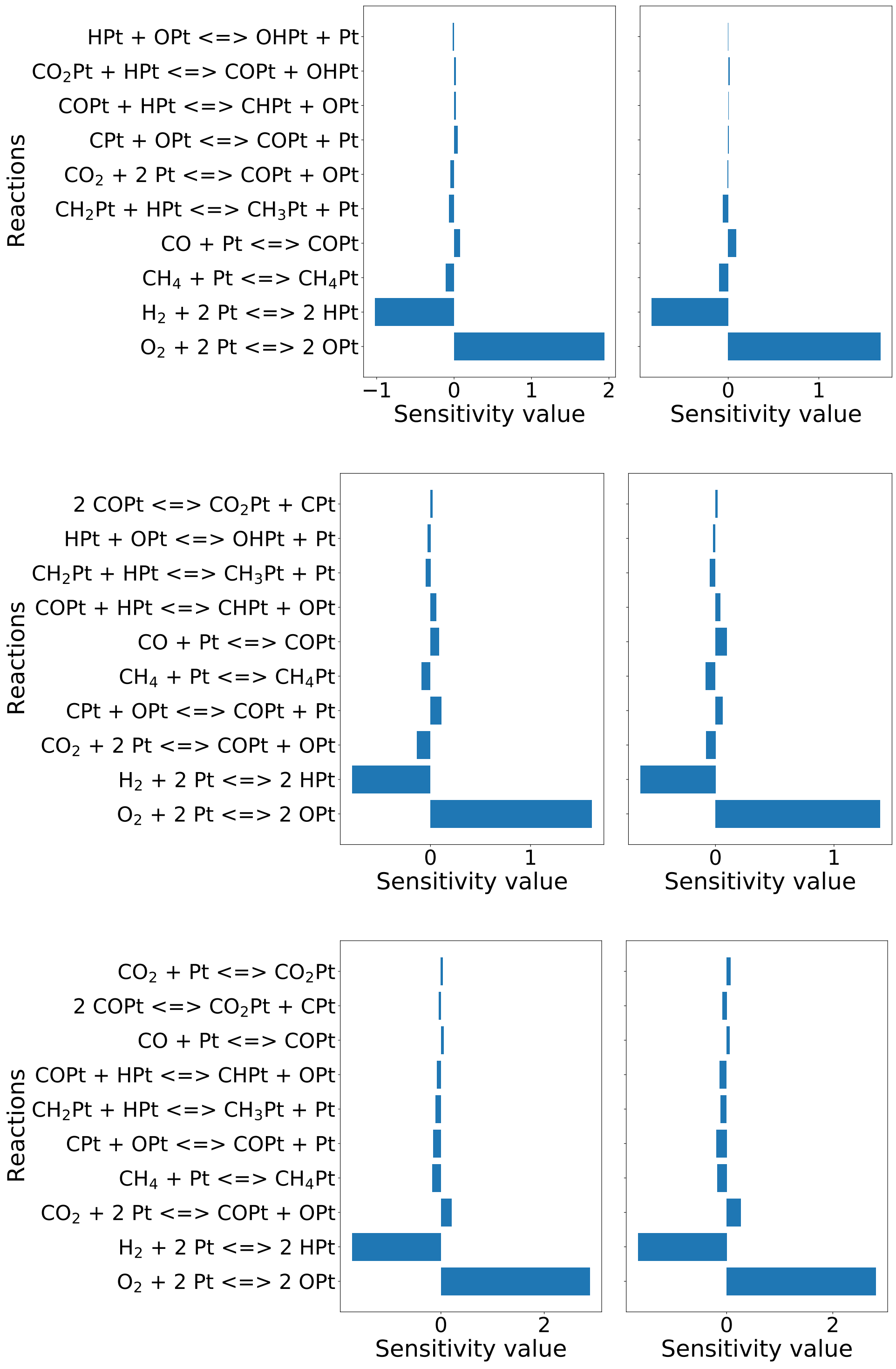

Figure S4: Kinetic sensitivity of CH_4 conversion (top plot), synthesis gas yield (middle plot), and full oxidation (bottom plot) comparisons for the CMPO (left) and CMPO-BMA (right) base Pt(111) models at C/O=2.6

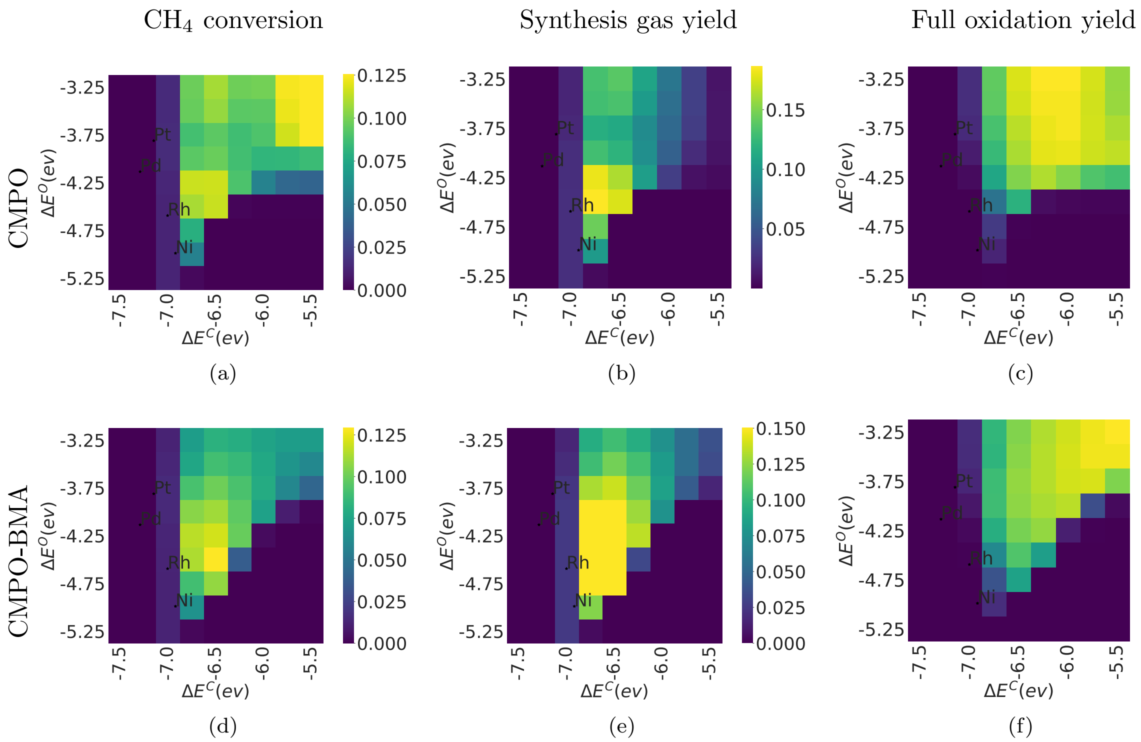

Figure S5: Comparison of CH_4 conversion, synthesis gas yield, and full oxidation yield at C/O=2.6 between CMPO ((a),(b),(c)) and CMPO-BMA ((d),(e),(f)) models. The y-axis represents the binding energy of atomic oxygen, and the x-axis represents the binding energy of atomic carbon, each pixel represents a hypothetical metal interface

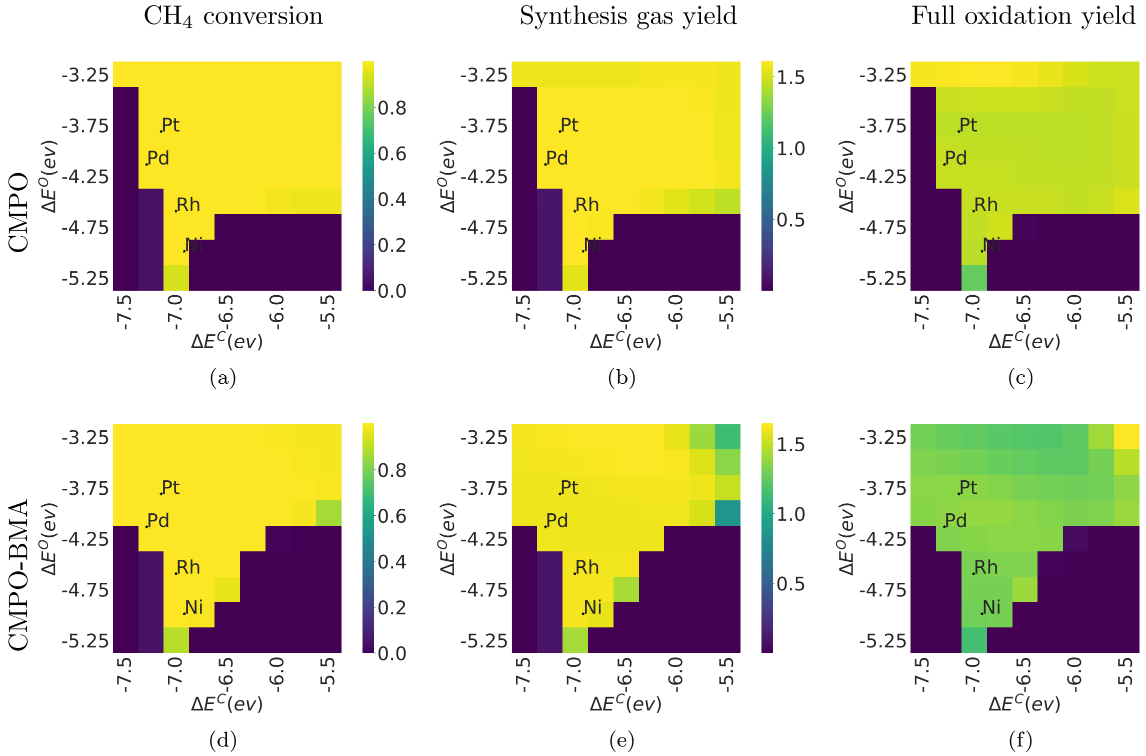

Figure S6: Comparison of CH_4 conversion, synthesis gas yield, and full oxidation yield at C/O=0.6 at the end of PFR between CMPO ((a),(b),(c)) and CMPO-BMA ((d),(e),(f)) models over 81 hypothetical metals. The y-axis represents the binding energy of atomic oxygen, and the x-axis represents the binding energy of atomic carbon, each pixel represents a hypothetical metal interface.

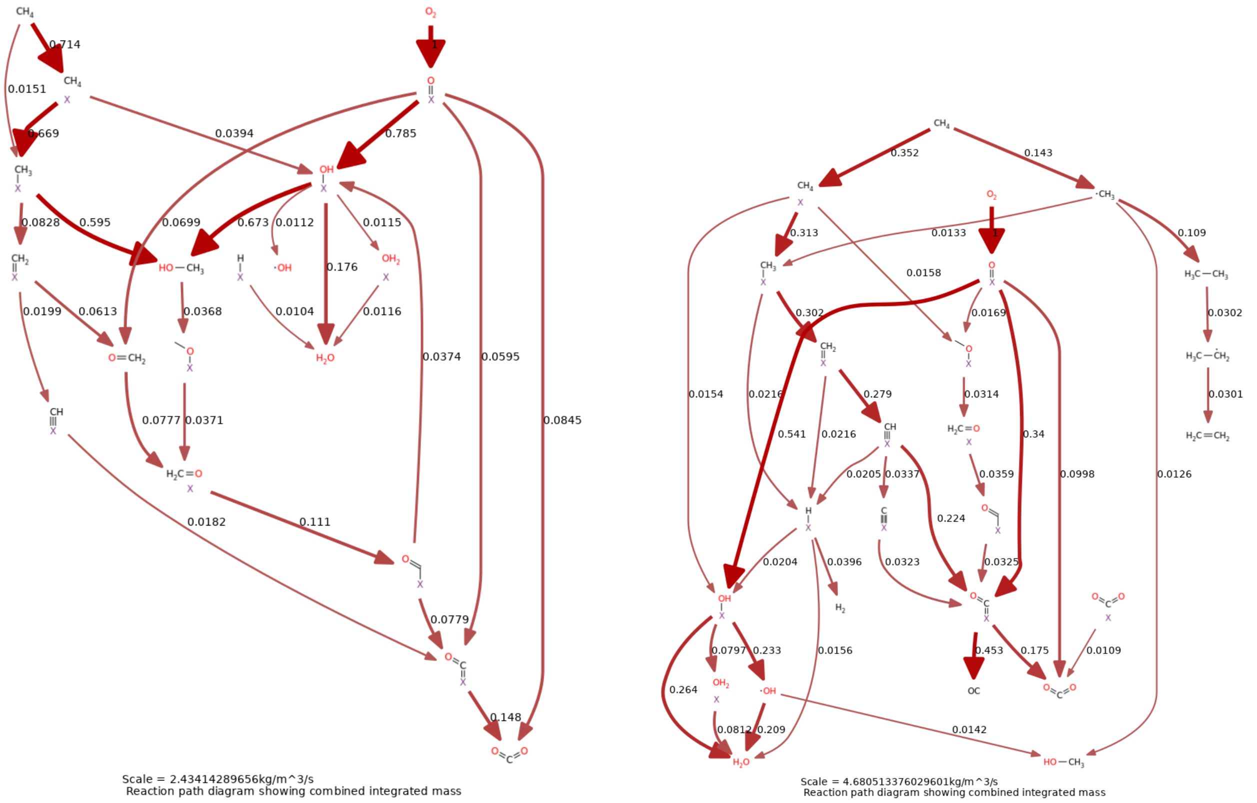

(a) Reaction path for CMPO model

(b) Reaction path for CMPO-BMA model

Figure S7: Comparison of the reaction paths in the CMPO and CMPO-BMA models on metal at ($\Delta E^O = -3.25$ eV, $\Delta E^C = -6.0$ eV). Line widths and labels indicate the net mass flux integrated from 0 to 1.045 cm in the reactor. The CMPO model makes lots of gas-phase methanol CH_3OH from the adsorbed CH_3*, whereas on the CMPO-BMA model adsorbed CH_3* continues to react via CH_2* and CH* to eventually make CO and CO_2.

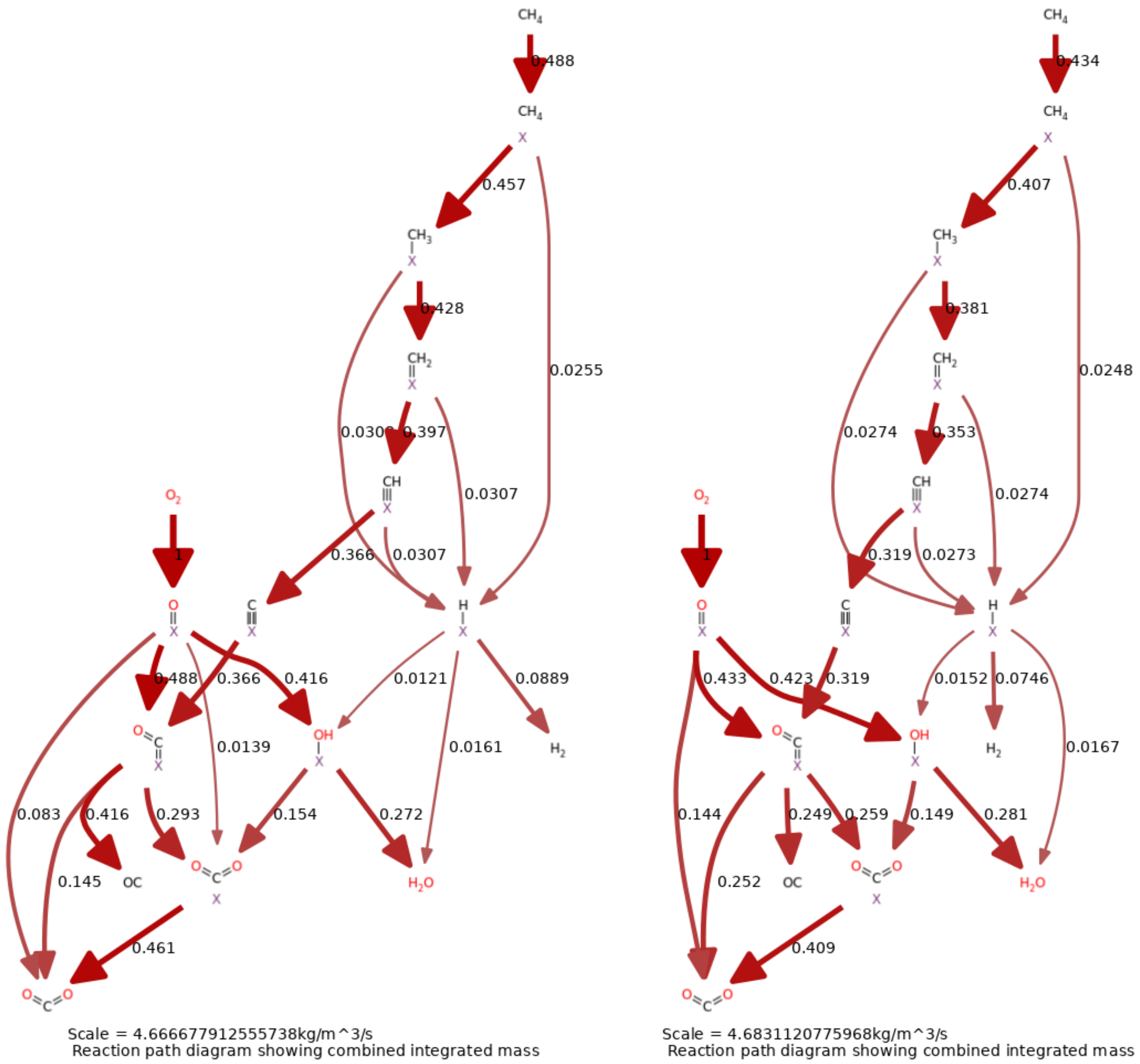

(a) Reaction path for CMPO model

(b) Reaction path for CMPO-BMA model

Figure S8: Comparison of the reaction paths in the CMPO and CMPO-BMA models on metal at ($\Delta E^O = -4.25$ eV, $\Delta E^C = -7.25$ eV). Line widths and labels indicate the net mass flux integrated from 0 to 1.045 cm in the reactor. They are quite similar.
